# Supplementary material for: Integrated multi-omics profiling uncovers miRNA-guided regulatory networks after spinal cord injury in rats
Source: Mol Ther Nucleic Acids. 2025 Oct 16;36(4):102746. doi: 10.1016/j.omtn.2025.102746 (PMC12617769; doi:10.1016/j.omtn.2025.102746)
Supplement: Document S1. Figures S1–S3 and Table S8 [file mmc1.pdf]

OMTN, Volume *36*

Supplemental information

Integrated multi-omics profiling uncovers miRNA-guided regulatory networks after spinal cord injury in rats

Ruslan A. Klassen, Sarka Chytilova, Ivan Arzhanov, Daniel Zucha, Eva Rohlova, Peter Androvic, Pavel Abaffy, Lucia Urdzikova-Machova, Mikael Kubista, Nataliya Romanyuk, and Lukas Valihrach

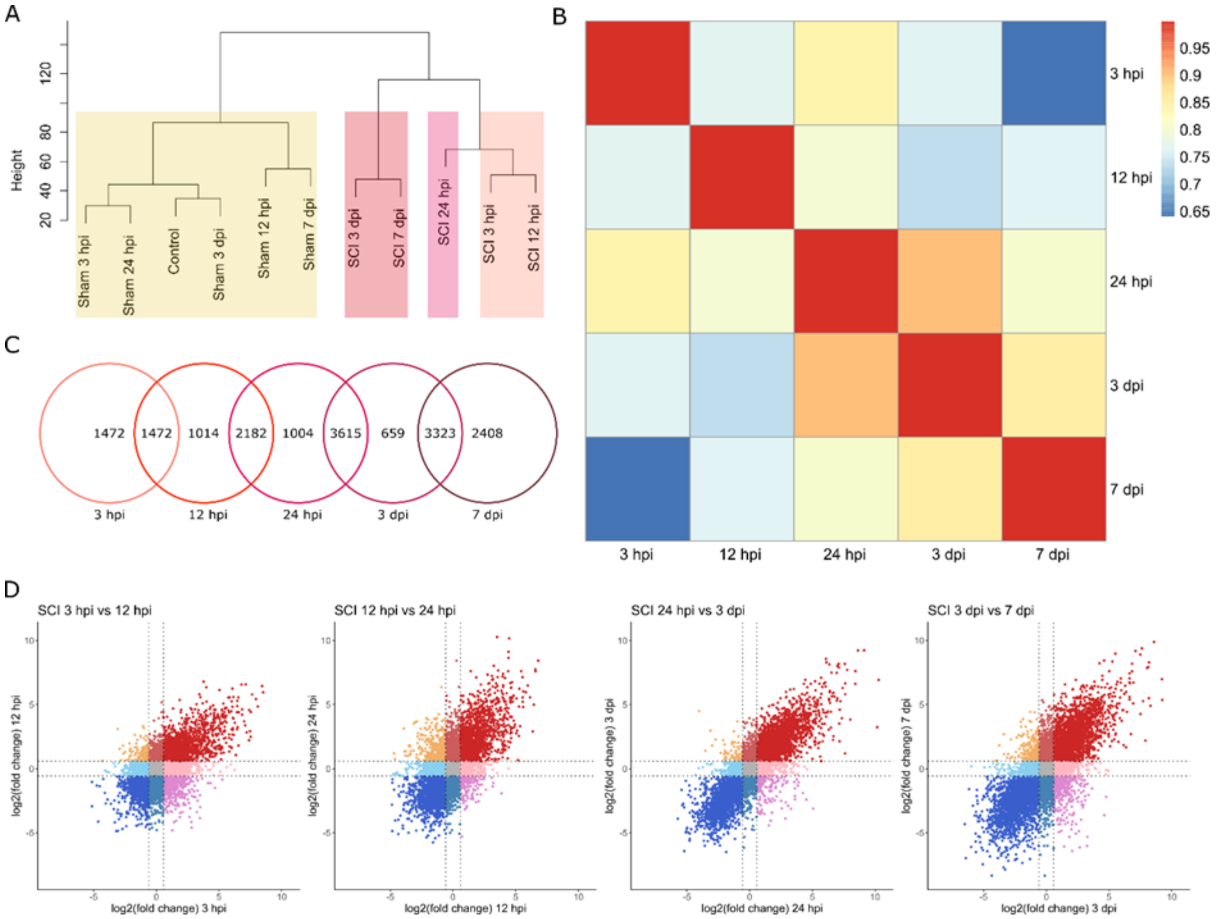

Figure S1: Sample similarity analysis.

A. Hierarchical clustering dendrogram of sample groups.

B. Pearson correlations of all DEGs between individual time points.

C. Overlap of DEGs between time points in ascending order from 3 hpi to 7 dpi ($p_{adj} < 0.05$ and $|log_2 FC| > 0.58$).

D. Scatterplot comparing log_2FC of DEGs between consecutive time points. Genes with $log_2FC > 0.58$ are highlighted in red, genes with $log_2FC < -0.58$ are highlighted in blue, and genes with log_2FC between -0.58 and 0.58 in grey. Genes that switch from up- to downregulated are highlighted in violet, and from down- to upregulated in orange.

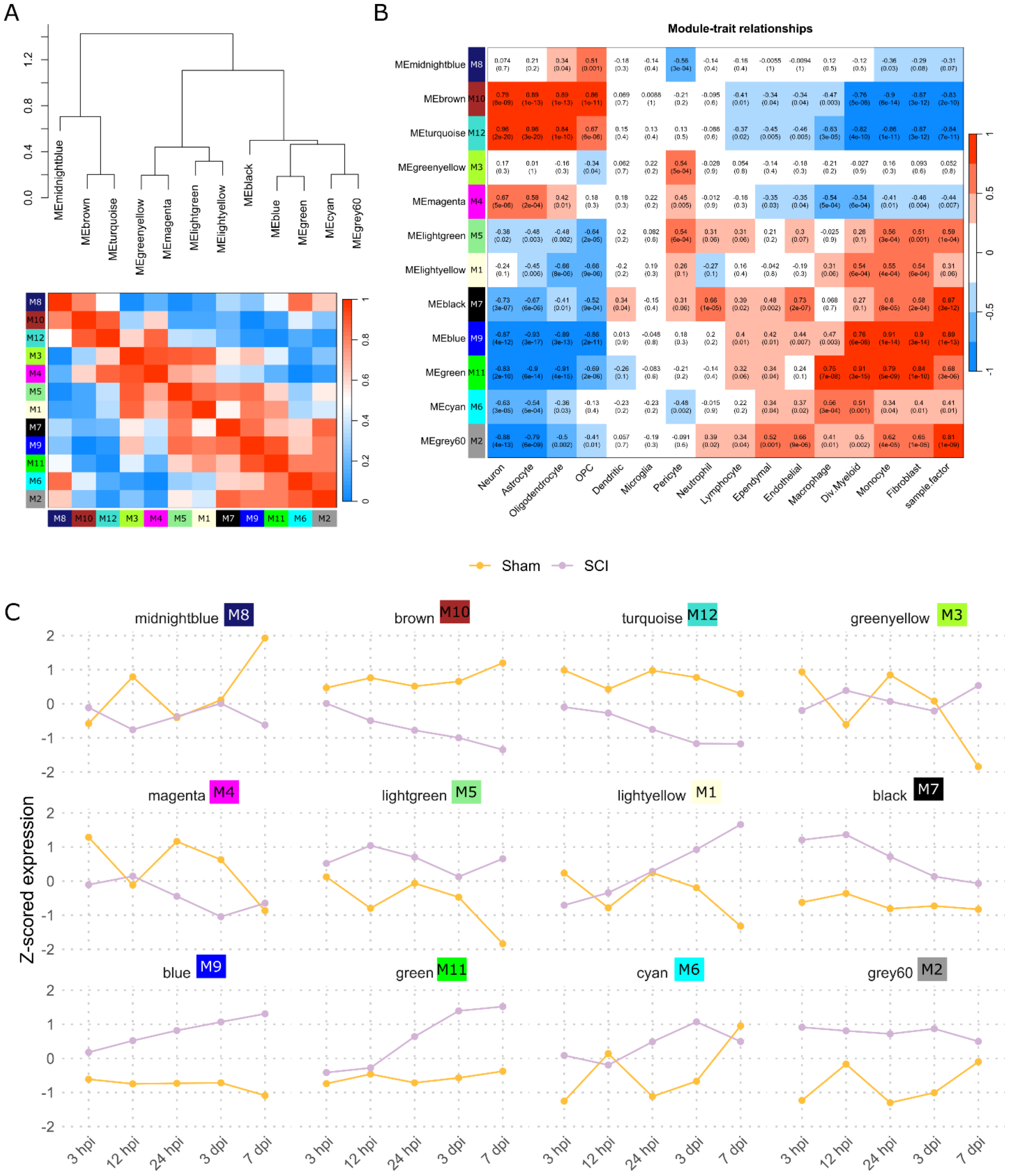

Figure S2: WGCNA module eigengene characterization.

A. Visualization of the eigengene network representing relationships among the modules. Hierarchical clustering dendrogram of modules eigengenes. The heatmap shows module eigengene adjacency.

B. Module-trait correlation analysis. Heatmap showing correlations between WGCNA module eigengenes and deconvoluted cell-specific profiles (see Fig. 4B) as well as injury status (sample.factor). Pearson correlation coefficients with p-values in brackets are displayed.

C. Mean expression profiles of all WGCNA modules.

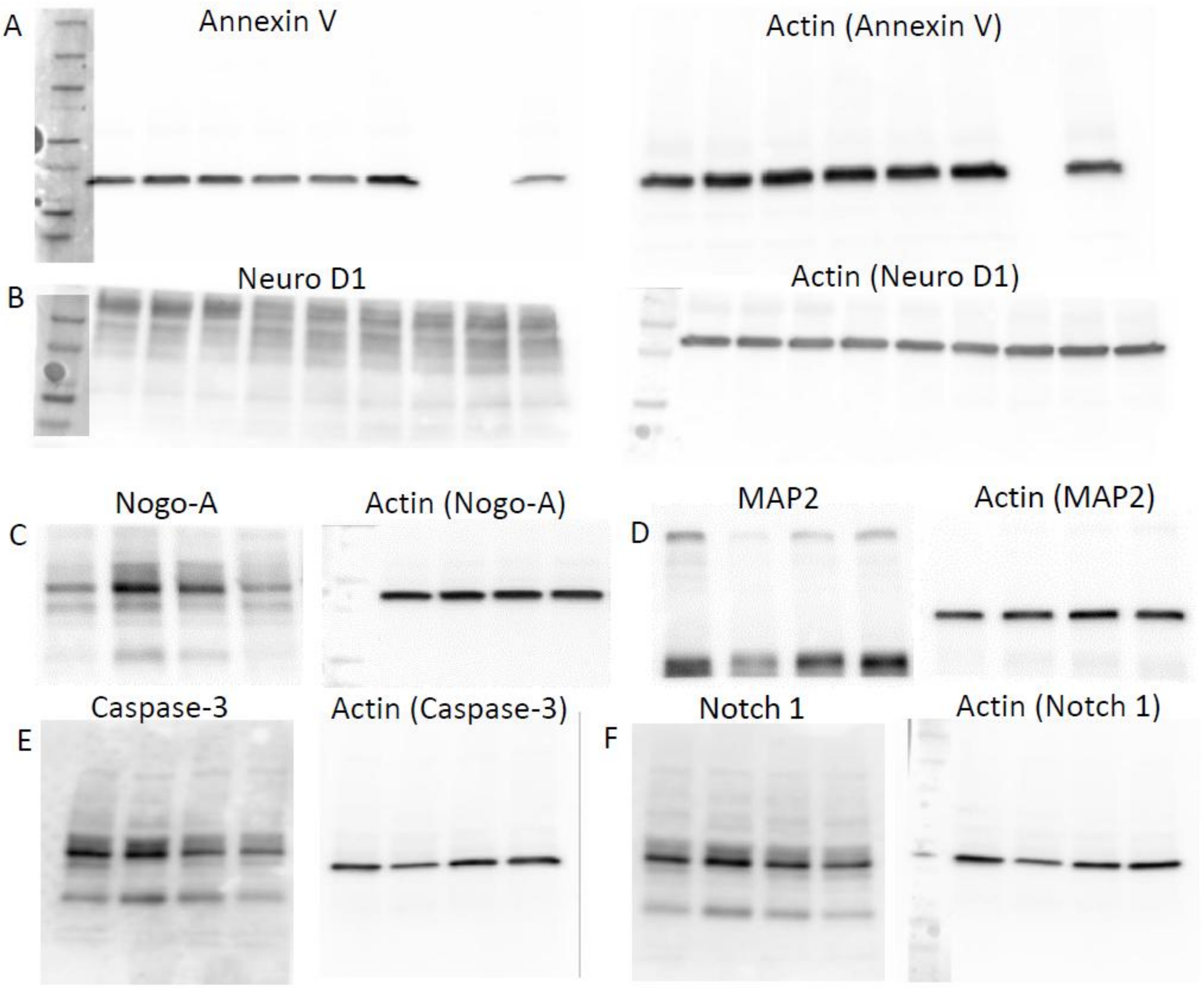

Figure S3: Raw images of blots presented in Figure 8.

Representative raw images of Western blots used to analyze levels of neuro-specific and apoptotic proteins shown in Figure 8. Actin was used as an endogenous control in each pair of images. Proteins analyzed: (A) Annexin V, (B) NeuroD1, (C) Nogo-A, (D) MAP2, (E) Caspase-3, and (F) Notch1. Nogo-A – neurite outgrowth inhibitor-A, MAP2 – microtubule-associated protein 2,

Caspase-3 – cysteine-aspartic acid protease-3, Notch1 – Neurogenic locus notch homolog protein 1.

Table S1: Differentially expressed mRNAs across time points post-injury.

Results of differential expression analysis at 3 hpi, 12 hpi, 24 hpi, 3 dpi, and 7 dpi. Each sheet corresponds to one time point. Columns include: baseMean (mean normalized counts), log2FoldChange, lfcSE (standard error of log2FC), stat (test statistic), pvalue, padj (adjusted p value, FDR), Ensembl gene ID, and symbol (gene symbol).

Table S2: Gene set enrichment analysis (GSEA) based on Gene Ontology terms

Significantly enriched biological processes, molecular functions, and cellular components identified from DEGs at each time point. Columns include ontology category (*ONTOLOGY*), GO *ID* and *Description*, *setSize*, *enrichmentScore*, *NES* (normalized enrichment score), *pvalue*, *p.adjust* (FDR), *qvalue*, *rank*, *leading_edge*, *core_enrichment*. Added columns: *time* (time point and direction, e.g., "3 hpi UP"), and *ratio* (number of genes contributing to enrichment relative to *setSize*).

Table S3: Over-representation analysis of curated gene sets.

ORA results of 118 gene sets compiled from 21 published studies, tested for overlap with DEGs from this study. Columns include: *ID*, *Description*, *GeneRatio* (DEG overlap/total DEGs), *BgRatio* (overlap/total background), *pvalue*, *p.adjust*, *qvalue*, *geneID*, *time* (time point and direction), and *ratio* (number of overlapping genes).

Table S4: Proteomics dataset (mass spectrometry).

Normalized and imputed protein abundance values. Columns represent samples, and rows represent individual proteins. The first column (*name*) indicates the protein symbol.

Table S5: WGCNA module eigengene characterization.

Summary of co-expression modules and enrichment results. Five spreadsheets are provided:

A. **Modules info** – module size and identifier (*ModuleColors*, number of genes, *module_name*).

B. **Modules info2** – gene membership and cross-species annotation (Ensembl ID, *ModuleColors*, module eigengene correlations, gene symbols/IDs across rat and mouse).

C. **DEGs enrichment** – ORA of module genes against DEGs from Table S1 (*ID*, *Description*, *GeneRatio*, *BgRatio*, *pvalue*, *p.adjust*, *qvalue*, *geneID*, module, *ratio*).

D. **Cell-type est** – ORA of module genes against CNS cell-type marker genes (Milich et al., 2021 re-analysis).

E. **ORA** – ORA of modules with GO Biological Process terms, including grouped categories (*group*).

TableS6 Differentially expressed miRNAs across time points post-injury.

Results of differential expression analysis for miRNAs at 3 hpi, 12 hpi, 24 hpi, 3 dpi, and 7 dpi. Columns include: baseMean, log2FoldChange, lfcSE, stat, pvalue, padj, and mirnaid.

Table S7: Interactome of regulatory relationships.

Comprehensive list of predicted and observed interactions between different molecular layers. Two spreadsheets are provided:

A. **miRNA–mRNA interactions** – calculated correlations between miRNA and mRNA expression (*miRNA*, *mRNA*, *pval*, *cor.coef*, *fdr*, *gene_symbol*, *ensembl_peptide_id*).

B. **mRNA–protein interactions** – calculated correlations between mRNA and protein expression (*mRNA*, *protein*, *pval*, *cor.coef*, *fdr*, *ensembl_peptide_id*).

Table S8. The list of the primary antibodies.

Antibodies name	Catalog number #	Molecular weight	Isotope	Dilution solution	Dilution
Annexin V	8555	30	Rabbit mAb	BSA	1:1000
Neuro D1	7019	49	Rabbit mAb	BSA	1:1000
NF-H	2836	180-220	Mouse mAb	BSA	1:800
Notch1	4380	120, 300	Rabbit mAb	BSA	1:1000
MAP2	4542	75, 82, 280	Rabbit mAb	BSA	1:1000
GFAP	12389	50	Rabbit mAb	BSA	1:400
Nogo-A	13401	180	Rabbit mAb	BSA	1:1000
Caspase-3	14220	35,19,17 kDa	Rabbit mAb	BSA	1:1000
β-Actin	A2228	42 kDa	Mouse mAb	TBST	1:2000
