# Supplementary material for: Integrated multi-omics profiling uncovers miRNA-guided regulatory networks after spinal cord injury in rats
Source: Mol Ther Nucleic Acids. 2025 Oct 16;36(4):102746. doi: 10.1016/j.omtn.2025.102746 (PMC12617769; doi:10.1016/j.omtn.2025.102746)
Supplement: Document S2. Article plus supplemental information [file mmc9.pdf]

Molecular Therapy
Nucleic Acids
Original Article

Integrated multi-omics profiling uncovers miRNA-guided regulatory networks after spinal cord injury in rats

Ruslan A. Klassen,[1,2] Sarka Chytilova,[3,4] Ivan Arzhanov,[5,6] Daniel Zucha,[1,4] Eva Rohlova,[7,8] Peter Androvic,[3] Pavel Abaffy,[1,7] Lucia Urdzikova-Machova,[5] Mikael Kubista,[3] Nataliya Romanyuk,[5] and Lukas Valihrach[1,7]

[1]Laboratory of Glial Biology and Omics Technologies, Institute of Biotechnology of the Czech Academy of Sciences – BIOCEV, 252 50 Vestec, Czech Republic; [2]Department of Biochemistry and Microbiology, Faculty of Food and Biochemical Technology, UCT Prague, 166 28 Prague, Czech Republic; [3]Laboratory of Gene Expression, Institute of Biotechnology of the Czech Academy of Sciences – BIOCEV, 252 50 Vestec, Czech Republic; [4]Department of Informatics and Chemistry, Faculty of Chemistry and Technology, UCT Prague, 166 28 Prague, Czech Republic; [5]Department of Neuroregeneration, Institute of Experimental Medicine of the Czech Academy of Sciences, Prague, Czech Republic; [6]Department of Neuroscience, 2nd Medical Faculty, Charles University, 150 06 Prague, Czech Republic; [7]GeneCore Facility, Institute of Biotechnology of the Czech Academy of Sciences – BIOCEV, 252 50 Vestec, Czech Republic; [8]Department of Anthropology and Human Genetics, Faculty of Science, Charles University, 128 43 Prague, Czech Republic

Spinal cord injury (SCI) is a debilitating condition with no effective treatment. The injury triggers a complex cascade of molecular and cellular events that drive both damage and repair processes. To explore these mechanisms, we performed a comprehensive multi-omics analysis in a rat compression model of SCI, focusing on the acute phase. Transcriptomic profiling revealed extensive gene dysregulation, highlighting early inflammation, neuronal death, and synaptic dysfunction, followed by the initiation of reparative processes. Cell type composition analysis showed a rapid infiltration of peripheral immune cells; activation of microglia; and loss of neurons, astrocytes, and oligodendrocytes. Importantly, we provide experimental support for predicted microRNA (miRNA)-mRNA-protein interactions, offering a foundation for further mechanistic studies. miRNA profiling uncovered a highly dysregulated miRNA landscape, with the miR-17~92 cluster emerging as a key regulator of neurogenesis, synaptic activity, and cell survival. Integrative miRNA-mRNA-protein analysis identified potential therapeutic targets, including miR-20a, whose inhibition *in vitro* supported neurogenesis and reduced apoptosis under oxidative stress. Our findings provide new insights into the molecular mechanisms of SCI and highlight miRNAs as potential targets for therapeutic intervention.

INTRODUCTION

Spinal cord injury (SCI) is characterized by acute damage to the spinal cord that causes temporary or permanent changes in its function. Every year, around one million people worldwide suffer SCI, with a substantial proportion leading to permanent loss of sensation and motor ability.[1] The acute care, hospitalization, and subsequent rehabilitation represent a major financial burden for patients as well as healthcare systems.[2] Despite tremendous efforts in research and numerous preclinical trials, there remains a lack of effective treatments.[3]

The complexity of SCI and our fragmented understanding of its pathophysiology contribute to this unsatisfactory situation. This has been improved by the advent of transcriptomic approaches and advanced computational methods, allowing for complex genome-wide characterization of the transcriptional landscape affected by SCI. Early microarray and RNA-sequencing (RNA-seq) studies provided important insights into the complex processes governing SCI progression.[4–7] More recently, single-cell transcriptomic methods have expanded our knowledge, revealing cellular heterogeneity and uncovering hidden pro-regenerative expression programs.[8–10]

MicroRNAs (miRNAs), a class of small RNA molecules, have emerged as important regulators of gene expression in various pathological conditions.[11] Their ability to modulate multiple transcripts simultaneously makes them promising candidates for future therapies. Indeed, manipulating miRNA levels via mimics or inhibitors has shown potential to ameliorate the progression of many diseases, including cancer, with some therapies currently in clinical trials.[12,13] Surprisingly, little attention has been paid to the characterization of miRNA-mRNA regulatory networks in SCI.[14,15] The few studies

Received 26 May 2025; accepted 14 October 2025;
https://doi.org/10.1016/j.omtn.2025.102746.

Correspondence: Nataliya Romanyuk, Department of Neuroregeneration, Institute of Experimental Medicine of the Czech Academy of Sciences, Videnska 1083, 142 20 Prague, Czech Republic.
E-mail: nataliya.romanyuk@iem.cas.cz
Correspondence: Lukas Valihrach, Laboratory of Glial Biology and Omics Technologies, Institute of Biotechnology of the Czech Academy of Sciences – BIOCEV, Prumyslova 595, 252 50 Vestec, Czech Republic.
E-mail: lukas.valihrach@ibt.cas.cz

© 2025 The Author(s). Published by Elsevier Inc. on behalf of The American Society of Gene and Cell Therapy. This is an open access article under the CC BY license (http://creativecommons.org/licenses/by/4.0/).

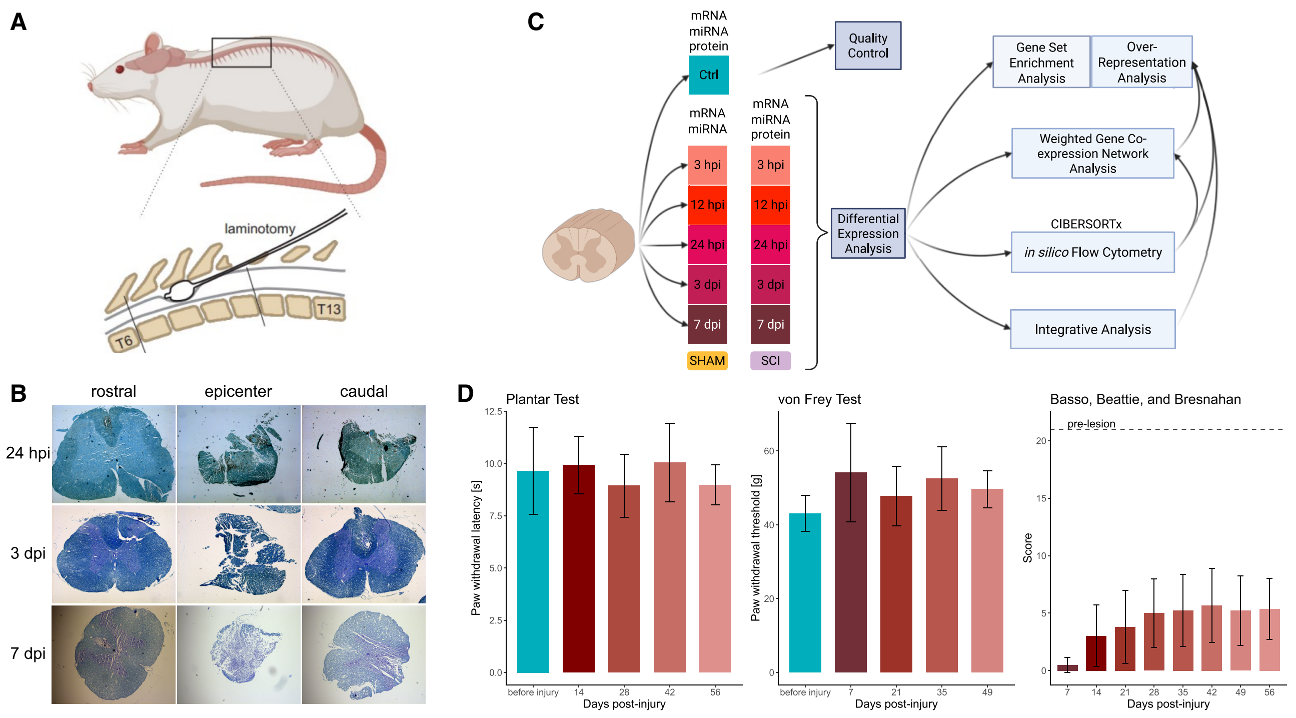

Figure 1. Experimental design

(A) Balloon compression model of spinal cord injury in rats (T7–T10). (B) Representative histological sections of the injured spinal cord at 1, 3, and 7 dpi. (C) Study design with key data analysis steps for multi-omics profiling post-SCI. (D) Behavioral assessments, including BBB locomotor scale, von Frey, and plantar tests. $p \geq 0.05$.

conducted have focused on a limited number of miRNA interactions or have used discordant miRNA and mRNA datasets.

In this study, we performed an in-depth characterization of the miRNA regulatory landscape during the acute phase of SCI using a rat compression model. We integrated miRNA, mRNA, and protein data to provide both a systems perspective and cell-type-specific context. Our analysis identified key miRNAs, such as those in the miR-17∼92 cluster, that regulate critical processes including neurogenesis and apoptosis. We validated some of these findings using *in vitro* models and demonstrated the potential of targeting miRNAs to influence SCI-related pathways, providing a foundation for future therapeutic strategies.

RESULTS

SCI in a rat compression model induces severe neurological deficits

To model SCI, we employed a balloon compression lesion in a rat model to induce a moderate injury to the thoracic spinal cord between the T7–T10 segments (Figure 1A). Histological analysis of coronal sections from the injured tissue revealed significant disruption of the tissue structure and volume reduction at the lesion epicenter at 1, 3, and 7 days post-injury (dpi). Additionally, secondary degenerative changes in tissue architecture were observed caudal to the lesion epicenter, whereas, rostral to the epicenter, the spinal cord tissue remained largely intact (Figure 1B).

We monitored selected locomotor and sensory functions of the injured rats for up to 56 dpi (Figure 1D). The Basso, Beattie, and Bresnahan (BBB) locomotion rating scale[16] indicated a significant impairment in motor function following SCI. Spontaneous recovery started after 7 dpi and continued until 28 dpi. However, the BBB score remained at least 3-fold lower than the pre-lesion levels throughout the observation period, indicating incomplete functional recovery. In contrast, sensory function, as assessed by the plantar test and von Frey test, was not significantly affected following SCI in the rats.

Thus, using a balloon compression lesion model in rats, we successfully established a stable SCI that resulted in significant motor deficits. This model provided a robust platform for subsequent detailed multi-omics analysis (Figure 1C), enabling comprehensive characterization of the molecular and cellular changes associated with SCI.

Acute phase of SCI involves inflammation, cell death, and loss of synaptic activity

To characterize the transcriptional changes during the acute phase of SCI, we performed RNA-seq analysis of spinal cord tissue in animals after laminectomy with injury and without injury (sham) and in control animals. We analyzed four samples per group at 3, 12, and 24 hours post-injury (hpi), as well as at 3 and 7 dpi. After excluding two outliers from the dataset, the remaining samples clustered into distinct groups based on time post-injury (Figure 2A). Samples

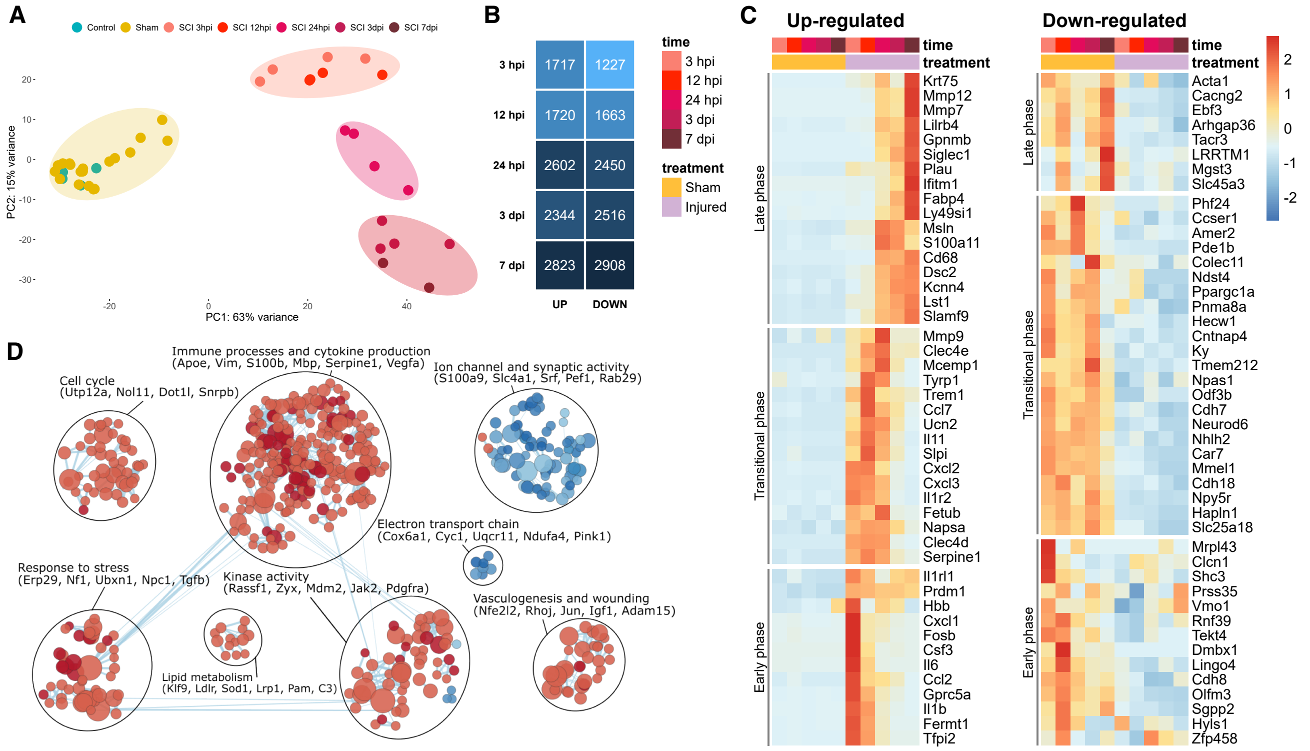

Figure 2. RNA-seq revealed a strong transcriptional response of the spinal cord to trauma

(A) Principal-component analysis (PCA) showing sample similarity based on mRNA expression profiles in low-dimensional space. See Figure S1 for additional details on sample similarity. (B) Total number of DEGs at 3, 12, and 24 hpi and 3 and 7 dpi (p_{adj} < 0.05 and $|\log_2 FC|$ > 0.58). See Table S1 for the complete list of DEGs, including expression levels and statistics. (C) Heatmap showing the nine most upregulated and downregulated genes at each time point (*Z*-scored normalized expression). See Table S1 for detailed information. (D) Enrichment map highlighting significantly enriched Gene Ontology (GO) terms (red circles, upregulated GO terms; blue, downregulated GO terms; size depends on size of GO term). Representative genes are indicated in brackets. See Table S2 for the full list of dysregulated processes.

from uninjured and sham animals formed the first cluster, while injured samples grouped into three clusters representing the early (3 and 12 hpi), transitional (24 hpi), and late (3 and 7 dpi) phases of the injury. This clustering pattern, confirmed by both hierarchical clustering and correlation analysis (Figures S1A and S1B), underscored the time-dependent nature of the transcriptional changes.

Since the number of dysregulated genes gives an indirect measure of the abnormalities caused by the trauma, we performed differential expression analysis (DEA) and determined the number of differentially expressed genes (DEGs). In total, we identified 4,918 upregulated genes and 4,655 downregulated genes after the injury (padj < 0.05 and |log2FC| > 0.58), indicating a strong transcriptional response of the tissue (Table S1). Examination of the DEG numbers at each time point showed relatively equal numbers of up- and downregulated genes throughout injury progression, with a peak during the transitional and late acute phases (Figure 2B). Notably, many of the dysregulated genes overlapped between consecutive time points, with only a minority being time point specific (Figures S1C and S1D; Table S1). This indicates the gradual nature of the transcriptional changes, with significant overlaps between the analyzed time points.

Next, we identified the most up- and downregulated DEGs at individual time points (Figure 2C). In the early acute phase (3 and 12 hpi), the most significant upregulation was observed in genes encoding cytokines and chemokines (e.g., *Il1b*, *Il6*, *Cxcl1*, *Cxcl2*, *Cxcl3*, and *Ccl2*), accompanied by the downregulation of Cdh8 and Shc3, whose roles in nervous tissue injury remain less well understood to date. The suppression of genes related to synaptic transmission (*Cntnap4*, *Npy5r*, and *Phf24*) persisted during the transitional phase (24 hpi) and was accompanied by an increase in the expression of *Mmp9*, *Clec4d*, *Mcemp1*, and *Slpi* and cytokine-related genes. The late stage of the acute phase (3 and 7 dpi) was characterized by a shift in trends, differing markedly from the early stages. Increased negative regulation of immune system processes (*Gpnmb*, *Lilrb4*, *Lst1*, and *Mmp12*) indicated a slowdown of inflammation and the initiation of neuroprotective factor production (*Lilrb4*, *Csf1*, and *Clec7a*).[17] In contrast, the most downregulated genes in the late phase were related to the postsynaptic specialization of the membrane (*Cacng2* and *LRRTM1*) and transmembrane transporter activity and lipid metabolism (*Slc45a3*), indicating the initiation of tissue remodeling processes that may contribute to subsequent regeneration.[18]

The gene-centric analysis revealed some of the key processes involved in the acute phase of SCI. To gain a more comprehensive view on the ongoing transcriptional changes, we applied gene set enrichment analysis (GSEA)[19] and visualized dysregulated biological processes in a network, collapsing closely related terms together (Figure 2D; Table S2). The analysis yielded several clusters, mostly composed of upregulated terms, representing the most dysregulated processes in the acute stage of SCI. These included activation of immune processes and cytokine production (*Apoe*, *Vim*, *S100b*, *Mbp*, *Serpine1*, and *Vegfa*), response to stress (*Erp29*, *Nf1*, *Ubxn1*, *Npc1*, and *Tgfb*), and kinase activity (*Rassf1*, *Zyx*, *Mdm2*, *Jak2*, and *Pdgfra*). These clusters were closely interconnected and contained the largest number of terms. Smaller groups of upregulated processes included cell cycle (*Utp12a*, *Nol11*, *Dot1l*, and *Snrpb*), vasculogenesis and wounding (*Nfe2l2*, *Rhoj*, *Jun*, *Igf1*, and *Adan15*), and lipid metabolism (*Klf9*, *Ldlr*, *Sod1*, *Lrp1*, *Pam*, and *C3*), highlighting cell proliferation and activation of reparative processes. The two groups of downregulated processes were related to ion channel and synaptic activity (*S100a9*, *Slc4a1*, *Srf*, *Pef1*, and *Rab29*) and the electron transport chain (*Cox6a1*, *Cyc1*, *Uqcr11*, *Ndufa4*, and *Pink1*), indicative of neuronal loss and reduced energy metabolism. Altogether, the analysis summarized the major changes undergoing during acute stage of SCI, mostly recapitulating results of single-gene based descriptive analysis.

In summary, the acute phase of SCI triggers a strong transcriptional response characterized by inflammation, immune activation, and synaptic disruption. Reparative processes emerge over time, but neuronal activity and energy metabolism remain impaired, reflecting the complex response to injury.

Temporal dynamics and shared injury mechanisms in acute SCI

To arrange the dysregulated processes in time, we repeated GSEA in individual time points and identified the most enriched terms (Figure 3B; Table S2). The first 3 hpi were characterized by positive enrichment of terms related to apoptosis, cell migration, proliferation, and inflammatory response, indicating immediate reaction of the tissue to the trauma. Simultaneously, negative enrichment of terms related to synaptic signaling and organization and cation and neurotransmitter transport highlighted detrimental effect of SCI on neurons. The negative effect on synaptic signaling peaked at 12 hpi and persisted at 3 dpi. Similarly, immune-related terms became dominant process at 12 hpi, peaked at 24 hpi, and decreased at 3 and 7 dpi. Interestingly, their closer inspection revealed involvement of different immune cells. While neutrophil-related terms appeared in the early acute phase, leukocyte activation was most prominent at 3 dpi. The enriched terms related to wound healing suggested the initiation of reparative processes at the latest time point. However, concurrent enrichment of immune related terms indicated that the resolving of the acute phase of the injury is not fully accomplished.

To place these findings in context, we compared our data with gene expression studies from various SCI models and other CNS injuries. We extracted 118 gene sets from 21 studies and calculated overlaps with the DEGs identified in our analysis (Table S3). We observed numerous significant overlaps (Figure 3A), which were consistent across different SCI models (compression, contusion, and transection) and species (*Mus musculus* and *Rattus norvegicus*), indicating a similar transcriptional response to spinal cord trauma.[9,20–22] Notably, a significant overlap with gene sets distinguishing severe versus moderate injury in a rat contusion model,[23] hinted at the severity of injury in our experimental model. We also detected overlaps with other CNS injury models, including permanent or transient middle cerebral artery occlusion, dorsal root ganglion axon injury, and systemic inflammation induced by lipopolysaccharide, suggesting that many transcriptional responses to trauma are shared across different CNS pathologies.

In conclusion, the transcriptional response to SCI shows early inflammation and synaptic dysfunction, followed by reparative processes. However, the persistence of immune activity suggests incomplete resolution of inflammation, potentially leading to prolonged pathology. These patterns are consistent with responses in other CNS injuries, indicating shared trauma mechanisms.

Cell type composition is substantially changed in the acute phase of SCI

The initial characterization of transcriptional changes introduced by SCI suggested the involvement of multiple cell types. To gain deeper insight into the cell type-specific responses, we employed the deconvolution algorithm CIBERSORTx.[24] Using a single-cell transcriptomic dataset as a reference, this tool estimates cell type proportions from bulk RNA-seq data. As a reference, we utilized a recent dataset from,[8] which investigated the uninjured and injured spinal cord at 1, 3, and 7 dpi using a mouse mid-thoracic contusion model. The selected dataset closely mirrored our experimental setting and included a broad spectrum of cell types, encompassing not only CNS cells but also peripheral immune cells (Figure 4A).

The deconvolution analysis revealed three prominent trends (Figure 4B). First, there was a dramatic decrease in the proportion of neurons, astrocytes, and cells of the oligodendroglial lineage, likely reflecting cell death. Notably, the rates of decline differed, indicating distinct sensitivities of these cell types to injury. The proportion of neurons dropped as early as 3 hpi; the major decrease in astrocytes occurred at 24 hpi; and oligodendroglial cells showed a slower decline, reaching a minimum at 3 and 7 dpi. Second, the analysis demonstrated a rapid and strong inflammatory response from both resident and peripheral immune cells. The first responders were neutrophils and dendritic cells (both peaking at 24 hpi), along with lymphocytes (maintaining a constant level of 2%–3% across all time points) and monocytes, whose proportions gradually increased until 3 and 7 dpi. At later time points, monocytes began to intermingle with macrophages, which together accounted for almost 30% of all cell types at the injury site. This inflammatory response was further amplified by microglia, which became activated at

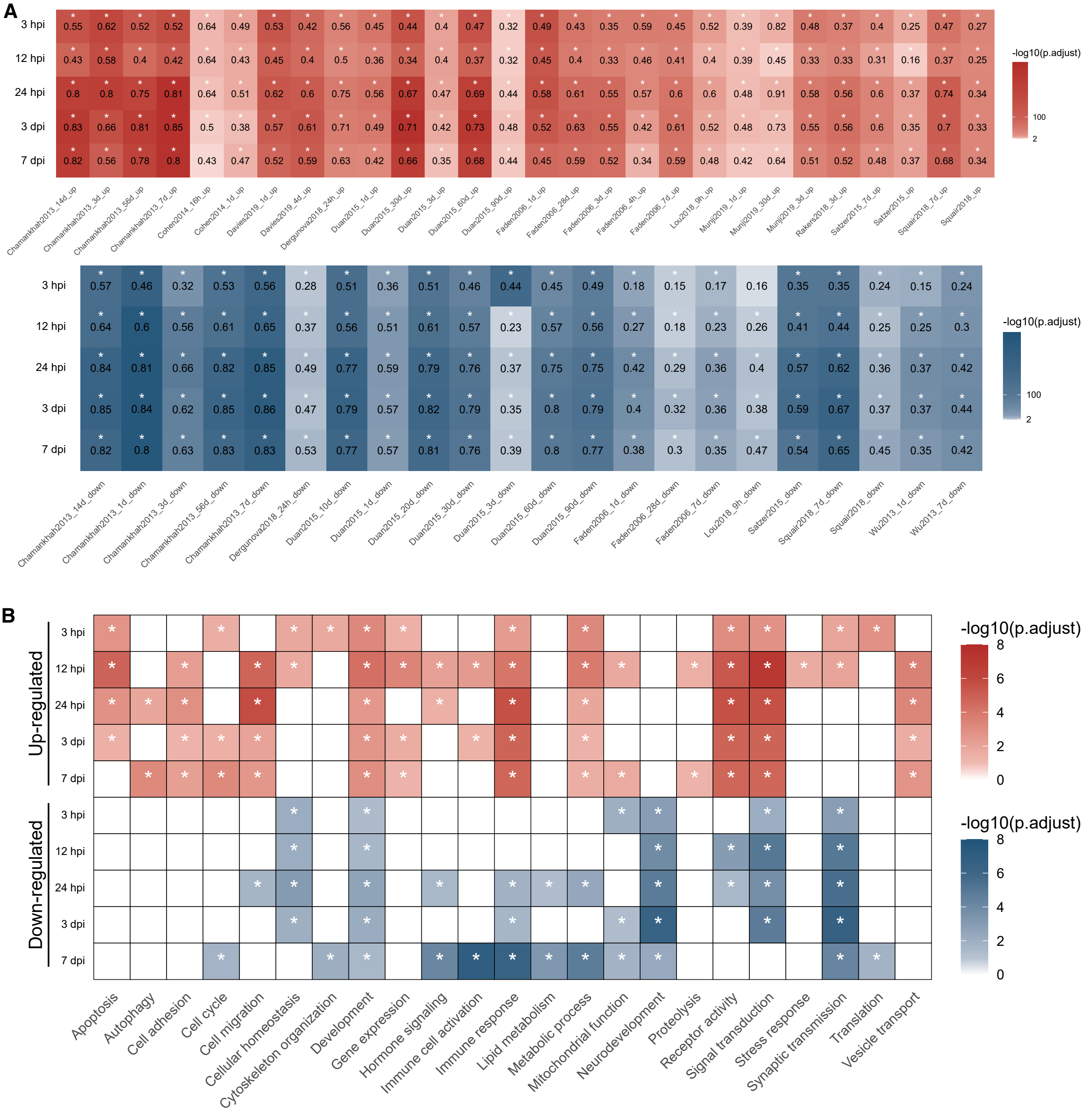

Figure 3. Characterization and meta-analysis of gene expression changes in SCI

(A) Size and significance of the overlap between DEGs in the current study and DEGs from selected studies. See Table S3 for the full list of studies used in the analysis. (B) The most significant GO term groups after GSEA dysregulated during the course of SCI ($p_{adj} < 0.05$, set size = 10–800). See Table S2 for the complete list of dysregulated processes.

24 hpi, peaking at 3 and 7 dpi. Lastly, we observed activation of ependymal and endothelial cells during the early acute and transitional stages (3–24 hpi), suggesting the restoration of the BBB, along with an increase in pericytes and fibroblasts, indicating fibrotic scar formation. Overall, the deconvolution analysis reflected major dysregulated processes (Figures 2D and 3B), supplementing them with cell type-specific information.

To cross-validate the deconvolution analysis, we employed a complementary approach by screening the lists of DEGs for enrichment

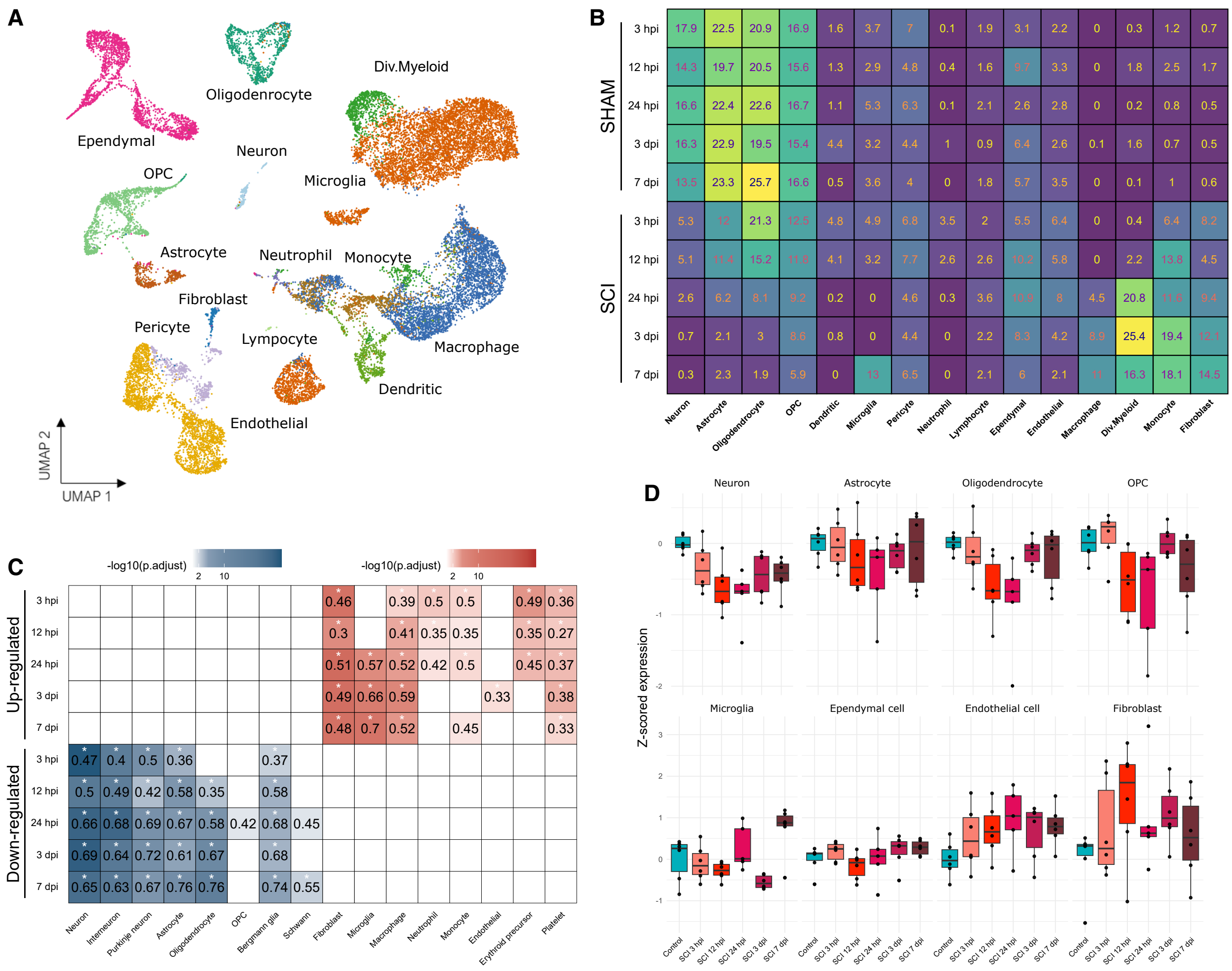

Figure 4. Changes in cell type composition during acute phase of SCI

(A) Major cell type identified for the re-analysis of a single-cell RNA-seq dataset from a mouse model of SCI.[8] OPCs, oligodendrocyte precursor cells. (B) Cell type proportion changes during SCI progression, estimated by computational deconvolution using CIBERSORTx and the single-cell reference from.[8] (C) *In silico* cross-validation of cell type changes using over-representation analysis (ORA) with marker genes from PanglaoDB. (D) MS validation of cell type proportion changes. *Z*-scored protein expression of selected markers from PanglaoDB for major cell populations. For details on MS analysis, see Table S4.

of marker genes defined by PanglaoDB, a database of over 1,000 single-cell transcriptomic studies.[25] The results mirrored those of the deconvolution analysis (Figure 4C). Marker genes for neurons, astrocytes, and oligodendroglial lineage cells were significantly enriched in the list of downregulated DEGs, indicating depletion of these cell types during SCI progression. We also observed a similar trend, with neurons appearing more sensitive than astrocytes and oligodendrocytes, while the latter two cell types exhibited a more pronounced decline during the later acute phases. As in the deconvolution analysis, significant enrichment of peripheral immune cell marker genes was detected in the list of upregulated DEGs. Neutrophils and platelets invaded the lesion immediately post-injury (3 hpi) and disappeared by 24 hpi, while the signal from monocytes and macrophages persisted throughout the acute phase. A delayed positive enrichment of microglial markers (24 hpi to 7 dpi) aligned with the observed rise in dividing myeloid cells at 24 hpi in the deconvolution analysis (Figures 4A and 4B). Finally, we observed positive enrichment of fibroblast and endothelial cell markers in the upregulated DEGs across all time points, highlighting their immediate and consistent involvement in the acute phase of SCI.

To further validate the *in silico* estimates of cell type proportion changes, we analyzed an independent set of samples using mass spectrometry (MS, Table S4). A total of 2,404 proteins were detected in control and SCI-treated animals. Using the PanglaoDB database, we identified markers for selected cell types and plotted their averaged *Z*-scored expression values during SCI progression (Figure 4D). Consistent with previous analyses, we observed a

decline in neuronal, astrocytic, and oligodendroglial protein expression. Microglial-specific protein expression declined during the early acute phase but increased in the late acute phase. Additionally, fibroblast, ependymal, and endothelial cell proteins were elevated at most time points compared to controls. Overall, the MS data recapitulated the trends in cell type proportion changes derived from RNA-seq data and validated the RNA-based computational estimates.

In summary, we documented substantial changes in cell type composition during the acute phase of experimental SCI. Key trends included the death of neurons, astrocytes, and oligodendrocytes, accompanied by the massive infiltration of peripheral immune cells and the activation of microglia, fibroblasts, and endothelial cells.

Network analysis provides systems perspective on the processes underlying SCI

Correlation network analysis is an efficient tool for analyzing large, high-dimensional datasets. To provide a comprehensive view of the transcriptional response of the injured spinal cord during the acute phase, we employed weighted gene co-expression network analysis (WGCNA). This method identifies modules of genes with similar expression profiles, suggesting that they may be involved in related biological processes. Moreover, these modules can be correlated with external traits, such as functional annotations or cell type enrichment, offering a system-level perspective on gene expression changes.[26]

Applying WGCNA to our dataset, we identified 12 modules of highly correlated genes, each showing distinct associations with the injury and different time profiles (Figures 2A–2C; Table S5). Four modules (black/M7, blue/M9, green/M11, and grey60/M2) were positively correlated with injury ($r > 0.6$, $p < 0.05$), indicating an increase in expression over time, while two modules (brown/M10 and turquoise/M12) showed negative correlation ($r < -0.6$, $p < 0.05$), reflecting a decrease in expression. We first examined the negatively correlated brown and turquoise modules (Figures 5A–5E). These modules shared a similar time-dependent decrease in expression and involved almost the same cell types and processes. They were enriched for neuron, astrocyte, oligodendrocyte, and oligodendrocyte precursor cell marker genes and downregulated DEGs during all acute phases of SCI (with the exception of 3 hpi for the brown module). These modules were involved in processes related to signal transduction, synaptic transmission, and neurodevelopment. Together, these two modules captured the major trends identified in previous analyses, including the extensive cell death of neurons, astrocytes, and oligodendrocytes (Figures 2C, 2D, 3B, and 4B–4D), leading to a sharp decline in locomotor function in the experimental animals (Figure 1D).

Next, we focused on the four modules that were positively correlated with injury (Figure 2B). These modules could be further grouped based on their time profiles: module with peak in the early acute phase (black), with a constant increase (blue and gray60), and module peaking in the late phase (green) (Figure 2C). The black module was highly (more than 20%) enriched for DEGs upregulated at 3–24 hpi, representing genes involved in the early acute response, particularly from peripheral immune cells such as neutrophils and monocytes. This response included processes related to cell migration and chemotaxis, indicating active recruitment of leukocytes to the injury site. Interestingly, this module also showed enrichment for endothelial and pericyte marker genes, alongside strong enrichment of the ERK1/2 (extracellular signal-regulated kinase 1/2) cascade, suggesting processes related to the re-establishment of the BBB and activation of vasculogenesis. The blue module represented the sustained activation of the immune system, driven primarily by peripheral immune cells, including neutrophils, monocytes, dendritic cells, and lymphocytes. This was reflected in the enrichment of biological processes related to leukocyte migration, proliferation, and adhesion. Finally, the green module was enriched for microglial marker genes, indicating activation and proliferation of resident CNS immune cells in the late acute phase. This response was coupled with persistent inflammation, mediated by lymphocytes and macrophages, which may exert cytotoxic effects on other cell types, as suggested by the enriched biological processes.

In summary, the WGCNA recapitulated the major trends observed in earlier analyses, including the death of neurons and supporting glial cells, along with the prominent multiphasic response of both peripheral and CNS-resident immune cells. Moreover, this analysis provided further refinement, enabling the assignment of specific functions to cell types in a time-dependent manner.

miRNA transcriptional profile is vastly dysregulated in the acute phase of SCI

miRNAs play an important regulatory role in a wide range of physiological and pathological conditions, and SCI is no exception. To gain insight into miRNA profiles affected by SCI, we performed small RNA-seq analysis using the same experimental setup and samples as the previous RNA-seq analysis. Since small RNA-seq is prone to various sources of bias, we used a circularization approach, which has been shown to effectively reduce major sources of bias and provide a more accurate representation of miRNAs.[27]

In total, we identified 315 miRNAs expressed across all time points (Table S6). Clustering analysis revealed a distinct separation between control and sham samples and those from the injured spinal cord (Figure 6A). The SCI samples further segregated into three clusters, with the first cluster containing samples from 3, 12, and 24 hpi and the remaining two clusters specific to samples from 3 to 7 dpi. Notably, this clustering differed from the previous mRNA-based analysis, which focused on protein-coding RNAs (Figures 2A and S1), indicating distinct temporal dynamics in the miRNA response. Next, we calculated differentially expressed miRNAs for each time point ($padj < 0.05$ and $|log2FC| > 0.58$; Figure 6B; Table S6). Consistent with the analysis of coding RNAs (Figure 2B), we observed a balanced number of upregulated and downregulated miRNAs, with the number increasing over time and peaking at 7 dpi (Figure 6B). Given the number of detected miRNAs and their capacity to regulate multiple targets simultaneously, the total number of

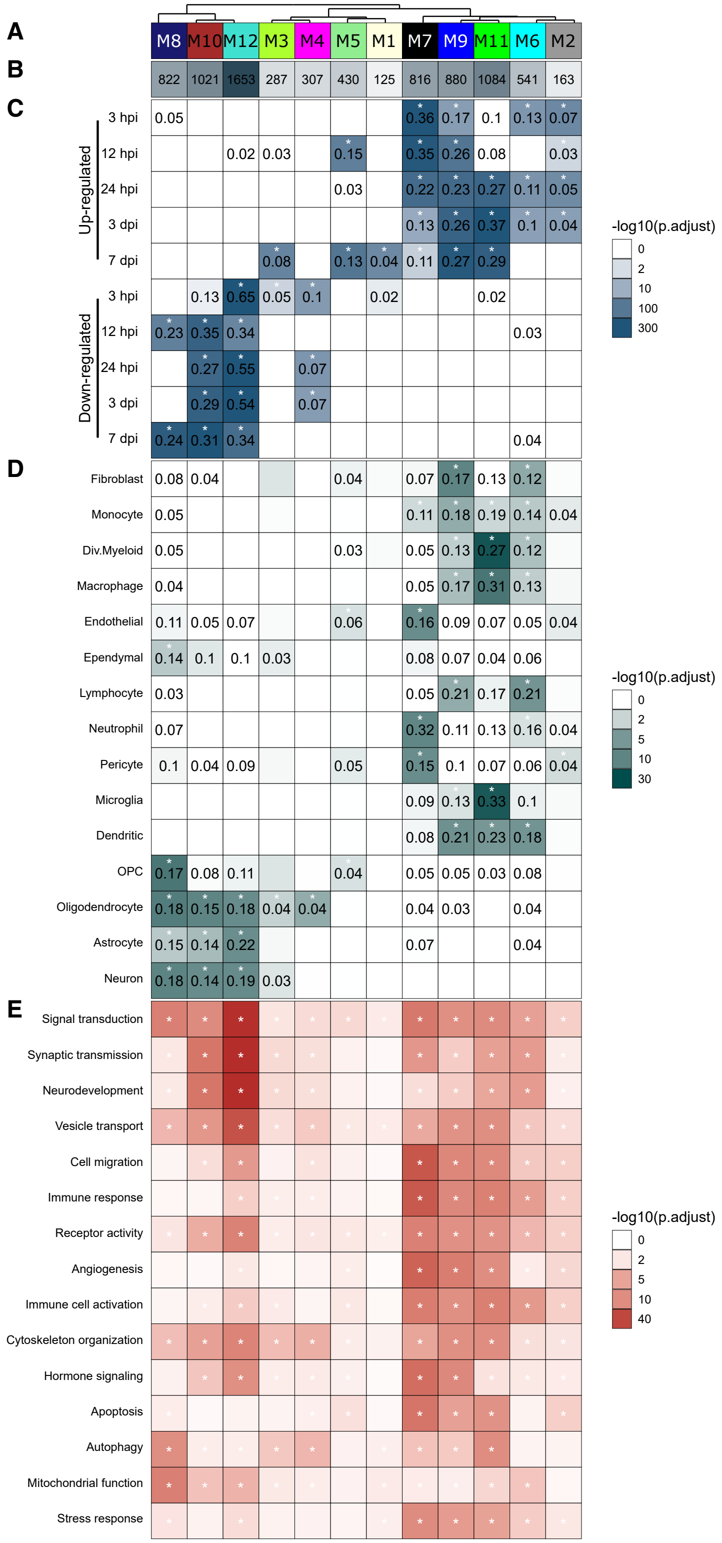

Figure 5. WGCNA identifies key cellular and molecular pathways driving SCI progression

(A) Color of modules (Figure S2A). (B) Number of genes in each module. (C) Representation of DEGs (Figure 2B) in WGCNA modules. (D) Enrichment of marker genes for major CNS cell populations as defined by re-analysis of Milich et al.[8] (E) Functional characterization of selected modules by ORA, listing the top dysregulated biological processes for each module. For details on WGCNA, see Figure S2 and Table S5.

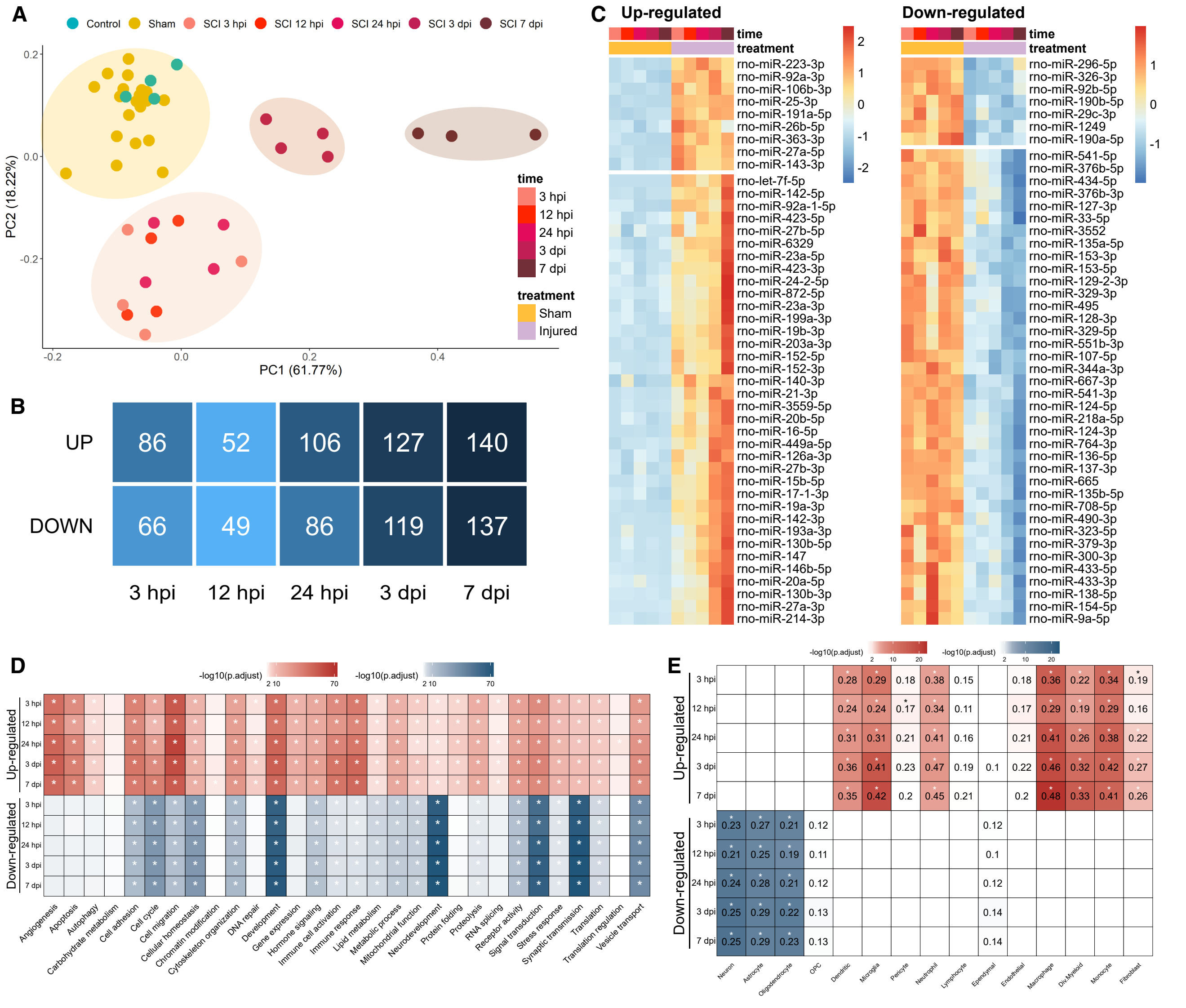

Figure 6. Dysregulated miRNA landscape following SCI

(A) PCA showing sample similarity based on miRNA expression profiles in low-dimensional space. (B) Total number of differentially expressed miRNAs at various time points post-injury (padj < 0.05 and |log2FC| > 0.58). (C) Heatmap showing the top differentially expressed miRNAs after SCI (sorted by fold change). See Table S6 for the complete list of differently expressed miRNAs. (D) ORA showing the most significant biological processes regulated by miRNAs. (E) ORA showing the major affected CNS cell populations by miRNAs.[8]

dysregulated miRNAs represents a major shift in the miRNA regulatory landscape.

To explore the response of individual miRNAs, we plotted the most dysregulated ones (sorted based on log-fold change) in a heatmap (Figure 6C). Most of these miRNAs were upregulated, suggesting a higher intensity of positive regulation in miRNA expression. Additionally, while the most downregulated miRNAs followed a uniform pattern of gradually decreasing expression, the upregulated miRNAs fell into three distinct clusters with different expression patterns. The first group peaked in the early acute phase, the second showed gradually increasing expression, and the third peaked in the late acute phase, suggesting different roles and/or origins for these miRNAs. Inspection of individual miRNAs from each cluster supported this notion.

The earliest response to injury is marked by the upregulation of miR-26a and miR-181c, along with the downregulation of miR-431 and miR-194. While the levels of these miRNAs tend to normalize within the first 12–24 hpi, several others remain persistently dysregulated throughout the entire observation period. Notably, miR-124 and miR-137 remain downregulated, whereas miR-21, miR-146a, and

miR-223 exhibit sustained upregulation. The late phase of post-injury response (3 and 7 dpi) is characterized by a notable decrease in the expression of miR-330, miR-335, miR-7a, and miR-7b. Concurrently, the expression of miR-126a, miR-339, miR-494, miR-497, miR-23b, and miR-125a is also upregulated, suggesting a complex and evolving regulatory network involved in the injury response and tissue remodeling.

To provide a broader perspective on miRNA-driven processes, we predicted mRNA targets of dysregulated miRNAs using miRWalk[28] and annotated their functions through over-representation analysis (ORA; Figure 6D). The same set of predicted targets was used to infer affected cell types (Figure 6E). This analysis closely mirrored our previous mRNA-based findings (Figures 3A, 4B–4D, and 5), revealing early activation of pathways such as vasculogenesis, inflammatory response, cytokine production, and cell migration, followed by extracellular matrix reorganization, cell adhesion, angiogenesis, and leukocyte invasion. Simultaneously, synapse-related processes were downregulated, along with negative enrichment for neuronal, astrocytic, and oligodendroglial markers. The mitogen-activated protein kinase (MAPK) cascade emerged as a central signaling axis orchestrating many of these processes, including proliferation, differentiation, motility, stress response, survival, and apoptosis.[29]

In summary, our miRNA analysis revealed a highly dysregulated regulatory landscape following SCI. The concordance between miRNA-predicted targets, affected pathways, and previously observed mRNA expression changes highlights the interdependence of post-transcriptional and transcriptional regulatory layers. Given the capacity of miRNAs to influence broad gene networks, these findings emphasize the potential for targeted miRNA-based interventions to modulate key pathological and reparative processes after SCI.

Integrative miRNA-mRNA-protein analysis identifies potential targets to manipulate SCI

To predict potential miRNAs for targeted intervention in our model of SCI, we performed an unsupervised multimodal analysis, integrating mRNA, miRNA, and protein data generated in this study. We applied a multistep correlation analysis, selecting significant negatively correlated miRNA-mRNA pairs ($r < -0.85$, padj < 0.05), representing the most potent interaction pairs. The resulting interaction network was then overlaid with protein data to highlight interactions where miRNA-mediated negative regulation was confirmed at the protein level.

In total, we identified 879 miRNA-mRNA-protein pairs (Table S7), which we visualized in an interaction network, referred to as the interactome (Figures 7A and 7B). The interactome consisted of a large cluster of upregulated miRNAs paired with downregulated mRNAs and proteins and a smaller cluster of downregulated miRNAs paired with upregulated mRNAs and proteins. The larger size of the upregulated miRNA cluster suggests active and complex regulation of synapse-related processes, while the smaller downregulated miRNA cluster points to tighter regulation of inflammatory and remodeling processes (Figures 6C–6E). Notably, the interactome included many miRNAs known to play a role in SCI pathogenesis, such as miR-21, miR-124, miR-155, and miR-146a, and have been implicated in key pathological processes including neuroinflammation, apoptosis, axonal regeneration, and glial scar formation. For instance, miR-21 is consistently upregulated after SCI and modulates PTEN (phosphatase and tensin homolog) and transforming growth factor β signaling pathways, contributing to both neuroprotection and astrocyte-mediated fibrotic scarring.[30] Similarly, miR-124, a neuron-specific miRNA, exerts anti-inflammatory effects by attenuating microglial activation and promoting neuronal differentiation via regulation of C/EBP-α and PU.1.[31] In contrast, miR-155 enhances the inflammatory response by targeting SOCS1, thereby activating the JAK/STAT pathway,[32] while miR-146a functions as a feedback inhibitor of the nuclear factor κB (NF-κB) pathway by downregulating TRAF6 and IRAK1, mitigating excessive cytokine production.[33]

To illustrate the information potential of the interactome, we focused on the miR-17∼92 cluster. Originally described as an oncogene, recent research has highlighted its role in angiogenesis and neurogenesis,[34] two processes critical for regeneration after injury. The miR-17∼92 cluster, consisting of eight miRNAs (miR-17-5p, miR-17-1-3p, miR-18a-5p, miR-19a-3p, miR-19b-3p, miR-20a-5p, miR-92a-3p, and miR-92a-1-5p), showed significant upregulation during the acute phase of SCI (padj < 0.05 and $|\log2FC| > 0.58$; Figure 5B; Table S6). Moreover, these miRNAs accounted for a major portion of the interactions in the interactome (Figures 7A and 7B), with about 698 target genes and 215 proteins (Table S7). We predicted the potential mRNA targets and annotated the associated biological processes (Figure 7D). The downregulated processes primarily involved synapse-related functions and axonogenesis, confirming the role of the miR-17∼92 cluster in neuronal plasticity following SCI.

Altogether, the integrative miRNA-mRNA-protein analysis generated a large interaction network, revealing the regulatory patterns following SCI and identifying novel miRNAs with previously unknown functions in SCI. By focusing on the miR-17∼92 cluster, we characterized its dysregulation and complex interactions within the interactome, highlighting it as a viable target for therapeutic manipulation.

Targeting miRNA-20a promotes neurogenesis and prevents apoptosis in *in vitro* models

To validate the potential of the miR-17∼92 cluster as a therapeutic target in SCI, we selected one of its less studied members, miR-20a-5p, and used an *in vitro* system to manipulate its expression. Our previous analysis revealed constant and significant upregulation of miR-20a-5p during the acute phase of SCI (Table S6). We used a miR-20a-5p inhibitor to assess its effects on neural plasticity and apoptosis in a culture of human-induced pluripotent stem cell-derived neural progenitors (iPSC-NPs).

To investigate the role of miR-20a-5p in neural stem cell (NSC) differentiation, we applied the inhibitor as a pretreatment before initiating iPSC-NP differentiation into a neuronal phenotype, followed

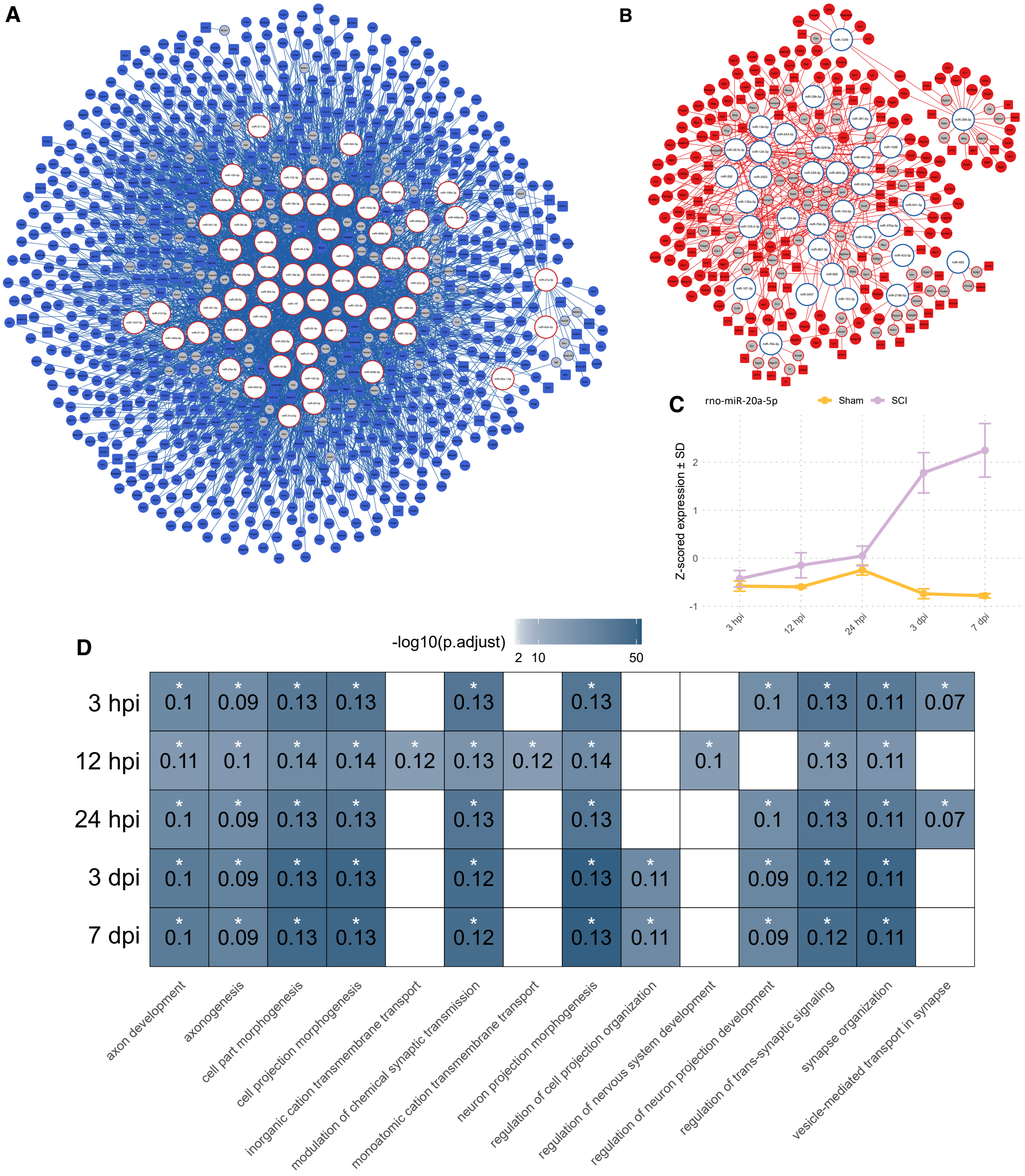

Figure 7. Interaction network of mRNA-miRNAs-protein dysregulation following SCI

(A and B) Interactome built on significantly dysregulated miRNAs (padj < 0.05 and |log2FC| > 0.58). miRNAs and mRNAs are shown as circles, while proteins are represented by rectangles. Blue indicates downregulation, and red indicates upregulation. mRNAs that did not meet the significance cutoff but connect significantly dysregulated miRNAs and proteins are shown in gray. See Table S7 for the complete list of interactions. (C) Average *Z*-scored expression with standard deviation (SD) of miR-20a-5p. (D) ORA showing downregulated biological processes regulated by the miR-17∼92 cluster.

by differentiation conditions for 10 days. Inhibition of miR-20a-5p led to a significant decrease (25.2% ± 0.2%, $p = 0.0083$) in the expression of the neuronal marker NEUROD1 compared to differentiated but untreated cells (Figure 8A). This suggests that miR-20a-5p inhibition promotes the suppression of NSC differentiation and helps maintain stemness properties, which are critical for repopulating the lesion.[35]

To further examine the effects of the miR-20a-5p inhibitor on apoptosis, we established an oxidative stress model using hydrogen

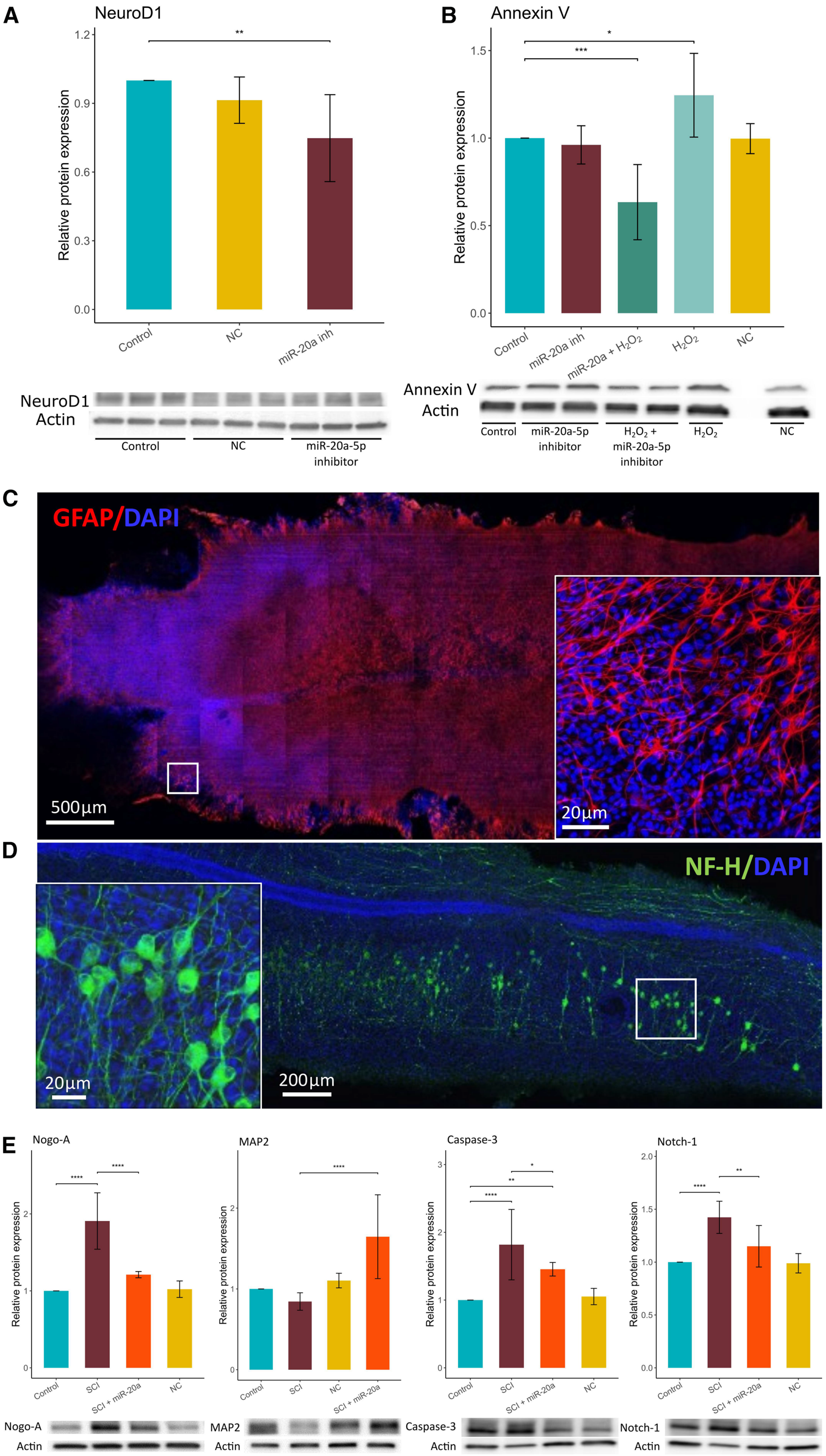

Figure 8. Manipulation of miRNA-20a levels in *in vitro* models

(A) Inhibition of miR-20a-5p modulates the expression of neurospecific maker NeuroD1 during iPSC-NPs differentiation. NC, negative control. (B) Inhibition of miR-20a-5p alleviates the negative effects of H_2O_2-induced oxidative stress by modulating Annexin V expression. (C) Representative histological image of a rat spinal cord longitudinal organotypic slice showing GFAP-positive astrocytes after 3 weeks in culture. GFAP, glial fibrillar acidic protein. (D) Immunocytochemical staining shows NF-H-positive neurons in a rat spinal cord longitudinal organotypic slice after 3 weeks in culture. NF-H, neurofilament heavy. (E) Western blot analysis revealed that inhibition of miR-20a-5p had a beneficial effect on the expression of Nogo-A, MAP2, caspase-3, and Notch1 proteins following lesion induction *in vitro* in rat spinal cord longitudinal organotypic slices. Nogo-A, neurite outgrowth inhibitor-A; MAP2, microtubule-associated protein 2; caspase-3, cysteine-aspartic acid protease-3; Notch1, neurogenic locus notch homolog protein 1. *$p < 0.05$; **$p < 0.01$; ***$p < 0.001$.

peroxide (H_2O_2). iPSC-NPs were treated with 100 nM H_2O_2 for 2 h, followed by miR-20a-5p inhibitor (50 nM) or scrambled miRNA (negative control, 50 nM) for 3 days (Figure 8B). H_2O_2 treatment caused a 24.5% ± 0.3% increase (p = 0.0177) in the expression of the early apoptotic marker Annexin V compared to untreated control cells (Figure 8B). However, treatment with the miR-20a-5p inhibitor after H_2O_2 exposure normalized Annexin V expression, fully compensating for the oxidative stress induced by H_2O_2. Treatment with the miR-20a-5p inhibitor alone, as well as H_2O_2 followed by scrambled miRNA, did not result in significant changes compared to control cells.

To further investigate the role of miR-20a-5p in spinal cord lesion development, we employed an *in vitro* model of SCI using longitudinal organotypic spinal cord slices (OTSs) from rats. Following preparation, OTSs were cultured for 10 days to allow stabilization of their metabolic activity (data not shown). During this period, the tissue preserved spinal cord-specific morphology and cellular composition, as evidenced by the presence of GFAP-positive astrocytes and NF-H-positive neurons with characteristic morphology (Figures 8C and 8D). After lesion induction, slices were treated with 100 nM miR-20a-5p inhibitor solution in the culture medium for 72 h. Protein expression analysis revealed that lesioning significantly increased Nogo-A (91.1% ± 36.7%, $p = 1.3 \times 10^{-10}$), Notch1 (42.4% ± 15.2%, $p = 2.0 \times 10^{-5}$), and caspase-3 (81.7 ± 52.0%, $p = 4.2 \times 10^{-7}$) levels, while MAP2 expression tended to decrease (25.6 ± 10.8%, $p = 7.9 \times 10^{-2}$) compared to control tissue samples (Figure 8E). Notably, inhibition of miR-20a-5p activity after injury restored the expression of these proteins to levels comparable to those observed in uninjured tissue.

In conclusion, using an *in vitro* model, we demonstrated the beneficial effects of manipulating miR-20a-5p, which was identified as dysregulated in our *in vivo* model of SCI. The positive effects include maintaining stemness properties and reducing apoptotic and neuroinflammation processes. These findings suggest that targeting miR-20a-5p could offer a promising therapeutic strategy for enhancing neural regeneration and preventing cell death after SCI.

DISCUSSION

The inherent complexity of SCI continues to drive the urgent pursuit of novel strategies to better understand the underlying pathological mechanisms. In this study, we present a comprehensive multi-omic analysis of SCI in a rat model, integrating gene, miRNA, and protein expression profiles during the acute phase of injury. Particular emphasis was placed on the analysis of a specific miRNA family to validate its involvement in post-injury pathophysiological processes.

Our analysis is based on a well-established rat spinal cord compression model, as supported by behavioral assessments of multiple animals in this and previous studies and evaluated at various time points post-injury.[36–41] Consistent transcriptomic data obtained from bulk RNA-seq of spinal cord tissue further substantiated the model. The sequencing results revealed pronounced pathological alterations, including downregulation of gene modules associated with synaptic transmission, increased expression of genes involved in cell death, and reactivation of the cell cycle—all hallmarks of acute SCI pathology.

The strength of our study lies in the generation of transcriptomic consensus signatures through the integration of miRNA, mRNA, and protein expression data, providing both a system-level perspective and cell-type-specific context. To the best of our knowledge, this is the first study to offer experimental confirmation of previously *in silico*-predicted interactions between specific miRNAs and their corresponding mRNA and protein targets. While *in vitro* and *in vivo* validation of many of the described changes remains a subject for future investigation, here we highlight several of the most significantly dysregulated miRNAs that warrant particular attention. For example, early down-regulated miRNAs such as miR-431 and miR-194 exhibit distinct roles in facilitating the initial injury response by alleviating inhibitory constraints on pathways crucial for inflammation, apoptosis, and neural repair. miR-431, a motor neuron-specific regulator, is significantly downregulated at 3 hpi, potentially promoting regenerative processes by releasing suppression on genes involved in neurite outgrowth and axonal extension.[42] Similarly, miR-194, a neuroprotective miRNA known to inhibit neuronal apoptosis via IGF1R regulation, is also downregulated at early time points (12 hpi), possibly reflecting a transient prioritization of pro-inflammatory and survival pathways over immediate neuroprotection.[43] miR-124 and miR-137, notably downregulated across all time points (from 3 hpi to 7 dpi), suggest prolonged suppression of key regulators of neuronal differentiation and inflammation modulation, potentially leading to impaired neurogenesis and sustained glial activation.[44,45]

In contrast, early upregulated miRNAs including miR-26a and miR-181c (both elevated at 3 hpi) contribute dynamically to injury responses. miR-26a plays an anti-inflammatory and neuroprotective role by targeting *TREM1* and downregulating the TLR4/MyD88/NF-κB signaling cascade, thereby reducing microglial activation and neuronal apoptosis.[46] In contrast, miR-181c exerts a deleterious effect by promoting apoptosis and inflammatory signaling through Bcl-2 suppression and upregulation of pro-inflammatory cytokines, exacerbating tissue damage during the acute phase.[47] Several miRNAs, including miR-21, miR-146a, and miR-223, are consistently upregulated at all measured time points, suggesting a sustained regulatory presence across both acute and chronic phases. miR-21 exhibits cell-type-specific temporal dynamics—appearing earlier in astrocytes and later in neurons—where it contributes to anti-apoptotic signaling and modulation of inflammatory responses.[45] miR-146a and miR-223, both known for their roles in immune regulation, likely act to buffer excessive inflammatory signaling throughout the SCI timeline.[33,48]

During the late phase (3 and 7 dpi), the miRNA expression profile shifts to reflect a transition toward chronic inflammation resolution, angiogenesis, and tissue remodeling. Downregulated miRNAs in this period include miR-330, miR-335, miR-7a, and miR-7b. miR-330

and miR-335, with known roles in dopaminergic signaling and neural progenitor cell proliferation, may contribute to impaired neuroregeneration when persistently suppressed.[49,50] Sustained downregulation of miR-7a and miR-7b (both anti-inflammatory miRNAs) suggests unresolved or prolonged inflammation that may hinder repair mechanisms.[49]

Conversely, several miRNAs are upregulated during the same late phase, including miR-126a, miR-339, miR-494, miR-497, miR-23b, and miR-125a. miR-126a and miR-339 act as key regulators of angiogenesis and endothelial repair through their interactions with VEGF and other endothelial signaling pathways, facilitating vascular remodeling and restoration of perfusion in injured tissue.[49,51] miR-494 and miR-497, involved in oxidative stress adaptation, target mitochondrial pathways to reduce reactive oxygen species (ROS)-mediated damage during prolonged recovery.[52] miR-23b and miR-125a contribute to immune resolution by modulating macrophage polarization and cytokine expression, supporting the transition from inflammation to tissue repair[49]

As discussed earlier, among the hundreds of miRNAs dysregulated following SCI, several dozen play critical roles in orchestrating complex gene networks involved in neurogenesis, synaptic plasticity, and cell survival. Notably, the miR-17∼92 cluster has attracted considerable interest due to its multifaceted involvement in NSC function and its potential implications in neurological disorders and injury. In our study, we focused specifically on this cluster, particularly miR-20a, and demonstrated its involvement in developmental and apoptotic processes in neural cells through *in vitro* analyses.

The role of miR-20a in SCI has been implicated following observations of its upregulation for at least one week post-injury. This finding has been confirmed by several microarray studies as well as by our results (Figure 7C).[53–55] Overall, miR-20a has been shown to play a crucial role in the pathophysiology of SCI. Jee et al. demonstrated that the abnormal expression of miR-20a can induce secondary injury in adult mice subjected to a transection model of SCI.[56] Mechanistically, miR-20a targets neurogenin 1 (Ngn1), a transcription factor involved in neuronal differentiation and specification. Ngn1 is key in maintaining cell survival, self-renewal, and neurogenesis in both the normal and injured spinal cord.[56] In this study, we also demonstrate that manipulating the level of miR-20a during NSC differentiation significantly influences the expression of the transcription factor NeuroD1 (Figure 8A). NeuroD1 is not only critical for the survival and differentiation of adult-born granule neurons but also for their maturation and integration into the neuronal circuitry.[57] Additionally, it has been shown that manipulating NeuroD1 facilitates the conversion of astrocytes to neurons in a rat model of SCI.[58] Our proteomic and RNA-seq data demonstrated that NeuroD1 was downregulated in the time course after experimental SCI.

Our results demonstrate that the inhibition of miR-20a can mitigate the negative effects of ROS in an *in vitro* model of oxidative stress (Figure 8B). Treatment with a miR-20a inhibitor following the application of H_2O_2 led to a decrease in the expression of Annexin V, an early apoptotic marker (Figure 8B), and an increase in the proportion of viable cells (Figure 8D). Although Annexin V is probably not a direct target of miR-20a, since its expression in control cells was not affected by the miR inhibitor, miR-20a has shown a beneficial effect on apoptotic processes in neural stem cells.

Nogo-A is a key negative regulator of axonal regeneration in the central nervous system, as its receptor is expressed only by limited classes of neurons.[59] Increasing evidence indicates that the lack of nerve regeneration after SCI is associated with elevated expression of myelin-associated inhibitory molecules.[60,61] In our *in vitro* model of SCI, we demonstrated that inhibition of miR-20a-5p significantly reduced Nogo-A protein levels (Figure 8E), which were otherwise strongly increased after injury. These findings suggest that miR-20a-5p may act as a modulator of axonal outgrowth following nervous tissue injury.

Notch1 plays a multifaceted role in SCI, influencing NSC behavior, neuronal differentiation, and functional recovery.[62,63] Inhibition of Notch1 signaling has been shown to have beneficial effects in various contexts of SCI. For example, genetic knockdown or pharmacological inhibition of Notch1 can reprogram reactive astrocytes into neurons within the injured spinal cord, leading to the generation of neuroblasts and mature neurons.[63] Moreover, suppression of Notch1 signaling promotes differentiation of neural stem cells into neurons, contributing to tissue repair and functional recovery.[64] Consistent with these findings, we observed a significant downregulation of Notch1 protein levels (Figure 8E) in our *in vitro* SCI model upon inhibition of miR-20a-5p.

A similar effect was observed for the pro-apoptotic protein caspase-3. Caspase-3 is a central mediator of apoptosis and plays a critical role in secondary cell death following SCI. It is rapidly upregulated and activated in neurons and oligodendroglia, leading to extensive programmed cell death in both gray and white matter.[65,66] In our study, inhibition of miR-20a-5p significantly reduced caspase-3 expression (Figure 8E), indicating its potential role in mitigating apoptosis associated with SCI.

In contrast to these proteins, MAP2, a key component of the dendritic cytoskeleton, undergoes a marked decrease in expression after SCI, correlating with dendritic degeneration and cytoskeletal destabilization. This reduction contributes to neuronal dysfunction and impaired recovery.[67,68] In our model, modulation of miR-20a-5p levels increased MAP2 expression (Figure 8E) following injury, suggesting a protective effect.

Taken together, our findings demonstrate that inhibition of miR-20a-5p influences the expression of multiple proteins that are critically involved in SCI pathology. While these proteins are not direct targets of miR-20a-5p, its modulation exerts a broad regulatory effect that may offer therapeutic potential for mitigating several aspects of SCI.

In conclusion, this study provides an in-depth multi-omic investigation of SCI during its acute phase, uncovering a complex and dynamic landscape of molecular alterations at the gene, miRNA, and protein levels. Through the integration of transcriptomic and proteomic data, we confirmed the activation of hallmark pathological pathways, including neuroinflammation, apoptosis, oxidative stress, and synaptic dysfunction. Particular emphasis was placed on elucidating the roles of specific miRNAs—such as miR-20a and the miR-17∼92 cluster—which demonstrated significant temporal and functional relevance in modulating the SCI response. Importantly, we provide experimental support for predicted miRNA-mRNA-protein interactions, offering a foundation for further mechanistic studies. The differential expression patterns observed across time points underscore the necessity of temporally targeted interventions and suggest that miRNA-based therapeutics may hold promise in modulating key processes for functional recovery. Ultimately, this comprehensive dataset offers valuable insights into the molecular orchestration of SCI and establishes a robust platform for identifying novel biomarkers and therapeutic targets aimed at mitigating secondary injury and enhancing repair mechanisms in the injured spinal cord.

Limitations of the study

Our study has certain limitations. All experiments were performed in adult male rats to reduce variability and align with common practice in SCI research. However, it is increasingly recognized that sex influences injury responses, immune activation, and regenerative potential. The exclusion of females therefore limits the generalizability of our findings. Future studies including both sexes will be needed to strengthen translational relevance.

Our study identifies miR-20a as a promising therapeutic target and demonstrates its effects *in vitro*. However, without *in vivo* gain- or loss-of-function experiments, the therapeutic relevance remains limited. *In vivo* studies are essential to account for the complexity of the injured spinal cord environment, including immune responses and tissue interactions. Future work will therefore need to confirm both the efficacy and safety of miR-20a modulation *in vivo*.

We analyzed a 2-cm segment centered on the lesion epicenter to obtain sufficient material for molecular analyses. This approach inevitably included the lesion, perilesional areas, and relatively intact tissue. Such heterogeneity may dilute region-specific changes and obscure rostrocaudal gradients in gene expression. More precise sampling or spatially resolved methods could provide a clearer picture of local molecular dynamics.

MATERIALS AND METHODS

Animals

Adult male Wistar rats ($n = 64$) were used as an experimental model. The rats were obtained from the facility's breeding center (Physiological Institute of the Academy of Sciences of the Czech Republic, Prague, Czech Republic). Animals undergoing surgery weighed 300 ± 30 g and were 10 weeks old. Animals were maintained on a 12-h light/dark cycle with *ad libitum* access to food and water. Animals were housed in pairs in identical cages located in the same room and rack, under identical environmental conditions (temperature, humidity, and light/dark cycle). All animals originated from different litters, were of the same age and species at the start of the experiment, and were assigned to the same experimental group within each cage. Rats were randomly divided into experimental groups: SCI 3 hpi ($n = 4$), 12 hpi ($n = 4$), 24 hpi ($n = 4$), 3 dpi ($n = 4$), and 7 dpi ($n = 4$); time-corresponding shame-operated groups ($n = 4$ for each group); and an intact control group ($n = 4$); also 8 animals were used for behavioral tests, and 12 animals were used for immunohistochemical analysis.

All the experiments were performed in accordance with the European Communities Council Directive of 22nd of September 2010 (2010/63/EU) regarding the use of animals in research and were approved by the Ethics Committee of the Institute of Experimental Medicine CAS and subsequently by the Section Committee of Czech Academy of Sciences, Prague, Czech Republic (Project No. 54/2017, approved 14th of July 2017). All efforts were made to minimize both the suffering and the number of animals used.

SCI and tissue collection

A balloon compression lesion was performed as described by Urdzikova et al. In brief, the animals were anesthetized with 3% isoflurane (flow rate of 0.3 L/min, Forane; Abbott Laboratories, Queenborough, UK) in a combination with buprenorphine (Vetergesic Multidose for cats and dogs 0.1 mg/kg, Ceva Santé Ani-male, France). The rat's rectal temperature was kept throughout the surgery at 37°C with a heating pad to avoid hyperthermia.[69] The rats' back was shaved from C7 to T12 spinal level. Under sterile conditions, the skin was cut in the midline from T7 to T12. The soft tissue was removed, as well as the spinous processes of vertebrae T8 to T11. A 2F Fogarty catheter (Edwards Lifesciences, Irvine, CA, USA) was inserted into the epidural space and advanced cranially for 1 cm so that the center of the balloon rested at the T8 to T9 level of the spinal cord. The balloon was rapidly inflated with 15 μL of saline for 5 min. The catheter was then deflated and removed. The soft tissue and skin were sutured in anatomical layers, first muscles and then the skin. The surgery procedure was completed by administering antibiotics (ampicillin 60 mg/kg, 1x per day, for 5 days, Biotika, Czech Republic), and the animals were allowed to feed and drink ad libitum. After being returned to their cages, the rats were assisted in feeding and urination until they had recovered sufficiently to perform these functions on their own (approx. 2 weeks). Buprenorphine (Vetergesic Multidose for cats and dogs 0.05–0.1 mg/kg, as needed, Ceva Santé Animale, France) was used to prevent subsequent pain. The sham control animals underwent the identical procedure, including the insertion of the catheter, but without balloon inflation.

To collect spinal cord tissue for RNA extraction, animals were deeply anesthetized with ketamine (100 mg/kg) and xylazine (20 mg/kg). Their spines were rapidly dissected from body and placed on ice. The spines were opened, and a 2-cm-long segment of the spinal

cord was dissected between 1 cm cranial and 1 cm caudal to the injury epicenter. The collected tissue was placed into RNAlater (AM7020, Thermo Fisher Scientific, CA, USA) and stored at −80°C until analyzed.

To collect spinal cord tissue for histological analysis, animals were deeply anesthetized with ketamine (100 mg/kg) and xylazine (20 mg/kg). Their chest was opened, and transcardial perfusion was performed first with phosphate buffer until the clear solution flows and liver tissue was discolored as visual control of the quality of the perfusion. Then, the phosphate buffer was replaced with a 4% solution of paraformaldehyde (PFA) in phosphate buffer (250 mL). A 2 cm long segment of the spinal cord between 1 cm cranial and 1 cm caudal to the epicenter of the injury was dissected. The collected tissue was placed in a PFA solution and postfixed at 4°C for another 24 h. The PFA was then replaced with phosphate buffer, and the tissue was subjected to further analysis.

Histological analysis

Twelve animals were used for histological analysis. They were sacrificed at 1, 3, and 7 dpi, four animals for each time point. Two cm long spinal cords around the spinal cord lesion were embedded in paraffin. Serial cross-sections (thickness 5 μm, interval 1 mm) were obtained. Cresyl violet and Luxol fast blue staining were used for visualization of lesion. For histological analysis, spinal cord tissue samples were excluded from further processing if they showed visible signs of hemorrhage, were damaged during extraction, displayed lesions that were excessively large or small, or originated from animals with atypical behavioral test results. The samples were observed and photographed with an Axioskop 2 plus microscope (Zeiss, Oberkochen, Germany).

Behavioral testing

The BBB open field test, originally described by BBB[16] was used to assess basic locomotor functions (joint movement, weight support, forelimb-hindlimb coordination, paw placement, and stability of the body). Eight rats were used for functional testing throughout 56 days of evaluation period. The rats were placed in an open field arena and scored on the range of 0–21 points (0 indicated complete lack of motor capability, and 21 movements indicated a healthy rat). The rats were pretrained before induction of the spinal cord lesion to adopt testing conditions and then tested once per week starting 7 dpi. Both hindlimbs of the animal were scored separately. The final BBB score for each rat is the average from the BBB score of the right and left hindlimb.

The plantar test was performed using the plantar test instrument (Ugo Basile, Italy). A radiant thermal stimulus was applied to the plantar surface of the paws, and the latency of the paw withdrawal response was measured. The animals were pretrained before spinal cord surgery and then tested every 2 weeks, starting at day 14 post-injury. Each paw was stimulated five times, and the mean value was calculated for each paw. Hyperalgesia, as a response to the thermal stimulus, was defined as a significant decrease in the withdrawal latency.

The electronic von Frey test was performed using IITC Inc., Life Science Instruments (Woodland Hills, CA, USA), as described previously.[70,71] Each rat was measured 2 times before spinal cord surgery to adapt to the testing conditions and to get a baseline withdrawal threshold and then every 2 weeks starting at 7 dpi. Each paw was tested separately by slowly raising the probe with rigid tips against the paw. Pressure was increased until nociceptive response or until the rat lifted up the paw, so it was not possible to go further with the tip. The value was recorded, and measurement repeated until 5 values were measured for each paw. Of the 5 values the lowest and the highest were deleted and the rest three were average.

For all behavioral tests animals were placed in the testing room at least 30 min before the test to adapt. The rats were then removed from their home cages and placed into an open field arena or plexiglass boxes of the testing instruments and acclimatized there for 15 min before testing.

RNA and small RNA-seq

Total RNA was extracted using AllPrep DNA/RNA/Protein Mini Kit (QIAGEN, Cat. No. 80004) according to the manufacturer's protocol. RNA quantity and purity were assessed using the NanoDrop 2000 spectrophotometer (Thermo Fisher Scientific), and RNA integrity was assessed using the Fragment Analyzer (Agilent). All samples had RQN > 7. RNA libraries were prepared using 500 ng total RNA with QuantSeq 3′ Library Prep Kit FWD (Lexogen, Cat. No. 015) according to manufacturer's protocol, using 15 cycles of amplification. Each sample was spiked by 0.6 μL of ERCC spike-in (c = 0.01x; Thermo Fisher Scientific, Cat. No. 4456740). Final libraries were quantified on the Qubit 2 fluorometer (Thermo Fisher Scientific) and Fragment Analyzer (Agilent) and sequenced on the NextSeq 500 high-output (Illumina) with 85 bp single-end reads. 1.8–4.7 million reads were obtained per library with a median of 2.8 million reads. Small RNA libraries were prepared using 300 ng total RNA with RealSeq-AC miRNA Library Kit for Illumina (Somagenics Cat. No. 500-00012) according to manufacturer's recommendation, using 14 cycles of amplification and 0.6x adapter concentration. Final libraries were quantified on the Qubit 2 fluorometer (Thermo Fisher Scientific) and Fragment Analyzer (Agilent) and sequenced on the NextSeq 500 high-output (Illumina) with 85 bp single-end reads. 0.2–2.1 million reads were obtained per library with a median of 1.1 million reads. Of note, the identical samples were used for the RNA and small RNA-seq.

RNA-seq and small RNA-seq data processing

RNA-seq data was processed as follows. Adaptor sequences and low-quality reads were removed using TrimmomaticSE v.0.36.[72] Reads mapping to mtDNA and rRNA were filtered out using SortMeRNA v2.1 with default parameters.[73] The remaining reads were aligned to genome reference Rnor 6.0 using STAR v.2.5.2b with default parameters.[74] Mapped reads were counted over ensembl

Rnor v.0.6.93 gene annotation using htseq-count with union mode for handling of overlapping reads.[75] Small RNA-seq data were processed as follows. Adaptor sequences, low-quality reads, and sequences shorter than 16 bp and longer than 28 bp were removed using cutadapt v.1.18.[76] Reads mapping to rRNA and UniVec were filtered out using SortMeRNA v.2.1 database and bowtie aligner with one mismatch allowed.[77] Reads mapping to genome reference Rnor 6.0 were further mapped using STAR with "end-to-end" mode allowing 5% mismatch to mature rat miRNA sequences from miRBase v.22.[78] Counting was performed with featureCounts function from Rsubread v.2.0.1, and only uniquely mapping reads were counted.

RNA-seq quality control and outlier removal

To ensure data quality and reproducibility, objective and *predefined exclusion criteria* were applied to RNA-seq samples prior to downstream analysis. Initial quality checks of raw sequencing reads were performed using FastQC v.0.11.9. For quantitative detection of outliers, two complementary approaches were used.

(1) *Principal-component analysis (PCA) with Mahalanobis distance*: Count data were normalized using log1p transformation, and genes with zero variance across samples were excluded. PCA was performed using the prcomp function in R, and Mahalanobis distances were calculated to identify multivariate outliers. Samples exceeding the 99.5th percentile of Mahalanobis distance from the PCA centroid were flagged as outliers.
(2) *Library size filtering via interquartile range (IQR)*: Total read counts per sample were calculated, and any sample falling outside *1.5× the IQR* from the first or third quartile was flagged.

Samples that met *both criteria* were excluded from downstream analysis. This two-step filtering was established *prior to analysis* and applied uniformly across all samples. As a result, *two samples from the 7 dpi group* were excluded due to consistently aberrant expression profiles and extreme library sizes.

Differential expression analysis

DEA of both mRNAs and miRNAs was performed using the DESeq2 R package v.1.26.0.[79] For mRNA, control samples were first compared against all sham-operated samples across time points to verify baseline expression stability. For miRNA, samples were annotated by condition (SCI, Sham, or Control), and only SCI and Sham groups were retained for downstream comparisons. Raw counts were normalized and filtered to remove lowly expressed features (average normalized counts < 5). For both mRNA and miRNA, separate DESeq2 analyses were conducted for each time point post-injury (3, 12, and 24 hpi and 3 and 7 dpi). The Wald test was used with a local dispersion fit, and differential expression was assessed for the contrast SCI vs. Sham at each time point. Genes and miRNAs with adjusted p values (padj) < 0.05 and absolute log2 fold change greater than 0.58 were considered significantly differentially expressed. Results are available as searchable tables in Table S1.

Enrichment analysis

Enrichment analysis was performed using ORA and GSEA.[80] ORA was calculated using custom script calculating the odds ratio (OR) and the significance of the overlap of the gene sets of interest with the Fisher's exact test. In the GSEA, a gene score was calculated for every gene using DESeq2 result as $-\log10(p_{adj})$ and assigned a positive or negative sign based on direction of regulation. Genes were ranked by their gene-scores and GSEA was run in a weighted preranked mode with 1,000 permutations. Gene sets were downloaded from http://download.baderlab.org/EM_Genesets/, and GSEA was run separately for three Gene Ontology (GO) categories (biological process – GOBP, molecular function – GOMF, and cellular component – GOCC). Only gene sets containing between 10 and 800 genes were considered. Cytoscape v.3.9.1 was used to visualize enriched gene sets as a network, where each node represents gene set and highly overlapping gene sets are connected with edges.[81]

Deconvolution

CibersortX is transcriptome deconvolution algorithm that uses a gene expression matrix of individual cell types as a reference, based on which it approximates the cellular composition of mixed sample by a linear support vector regression.[24] For the reference, we downloaded and in-house-reanalyzed published single-cell RNA-Seq dataset of injured mouse spinal cord[8] (GSE162610). Upon quality control, only the dataset's batch 2 count matrix was used, due to low transcript coverage in the other batches. Using the *Seurat* v.4.1.0 single-cell processing steps as a proxy,[82] the dataset was filtered for cells counting >200 unique transcripts, *SCTransform*-ed, and dimensions reduced with PCA (for 100 PCs). Top 30 PCs were selected optimal to reduce the data to UMAP visualization. The original cell cluster identity was retained using annotation of the authors. The reference matrix for deconvolution was built out of a gene list counting 23,624 genes with 400 randomly sampled cells per annotated cell-type cluster, with UMI counts as units. Calculation of single-cell RNA-seq signature matrix was done in default mode—quantile normalization disabled, min. expression of 0.75, replicates of 5, and sampling of 0.5. Imputation of cell fractions and group-mode expression were used in default settings, with S-mode batch correction enabled, quantile normalization disabled, and $n = 100$ permutations for significance analysis. Sample mixture file was submitted with unfiltered gene list (12,357 features), with UMI counts as units.

Weighted gene co-expression network analysis

Standard WGCNA procedure was followed to construct gene co-expression networks using the WGCNA R package v.1.71.[26] Filtered variance-stabilized (vst-transformed) expression data were used to calculate Pearson correlations between all gene pairs. Soft-thresholding was applied by raising correlation values to a power of 15 to emphasize strong correlations and suppress weak ones; this power was selected to approximate a scale-free network topology. A signed adjacency matrix was constructed and converted into a topological overlap matrix (TOM), with (1−TOM) used as a distance measure for hierarchical clustering. Gene modules were identified using the dynamic tree cut algorithm, with a minimum module size of 100

genes. Modules whose eigengenes (first principal component of module expression) were highly correlated (dissimilarity < 0.16) were merged. Each gene's module membership was calculated as the Pearson correlation between its expression and the corresponding module eigengene, and genes were assigned to the module where they showed the highest membership. Modules were further characterized by their overlap with DEGs, enrichment in major cell type markers, eigengene time profiles, and dominant dysregulated biological processes, using enrichment analysis for GO terms and cell-type markers.

Mass spectroscopy

Tissues were homogenized and lysed by boiling at 95°C for 10 min in 100 mM TEAB (tetraethylamonnium bromid) containing 2% SDC (sodium deoxycholate), 40 mM chloroacetamide, and 10 mM TCEP (tris(2-carboxyethyl)phosphine) and further sonicated (Bandelin Sonoplus Mini 20, MS 1.5). Protein concentration was determined using BCA (bicinchoninic acid) protein assay kit (23225, Thermo Fisher Scientific), and 30 μg of protein per sample was used for MS sample preparation. Samples were further processed using SP3 beads according to Hughes et al.[83] and digested with trypsin (trypsin/protein ration 1/30) reconstituted in 100 mM TEAB at 37°C overnight. After digestion, samples were acidified with TFA (trifluoroacetic acid) to 1% final concentration, and peptides were desalted using in-house-made stage tips packed with C18 disks (Empore) according to Rappsilber et al.[84] Nano Reversed phase columns (EASY-Spray column, 50 cm × 75 μm ID, PepMap C18, 2 μm particles, 100 Å pore size) were used for liquid chromatography-mass spectrometry (LC-MS) analysis. Mobile phase buffer A was composed of water and 0.1% formic acid. Mobile phase B was composed of acetonitrile and 0.1% formic acid. Samples were loaded onto the trap column (C18 PepMap100, 5 μm particle size, 300 μm × 5 mm, Thermo Fisher Scientific) for 4 min at 18 μL/min; loading buffer was composed of water, 2% acetonitrile, and 0.1% trifluoroacetic acid. Peptides were eluted with mobile phase B gradient from 4% to 35% B in 120 min. Eluting peptide cations were converted to gas-phase ions by electrospray ionization and analyzed on a Thermo Orbitrap Fusion (Q-OT-qIT, Thermo Fisher Scientific). Survey scans of peptide precursors from 350 to 1,400 m/z were performed in orbitrap at 120K resolution (at 200 m/z) with a 5×10^5 ion count target. Tandem MS was performed by isolation at 1.5 Th with the quadrupole, HCD fragmentation with normalized collision energy of 30, and rapid scan MS analysis in the ion trap. The MS2 ion count target was set to 10^4, and the max injection time was 35 ms. Only those precursors with charge state 2–6 were sampled for MS2. The dynamic exclusion duration was set to 45 s with a 10 ppm tolerance around the selected precursor and its isotopes. Monoisotopic precursor selection was turned on. The instrument was run in top speed mode with 2 s cycles.[85]

Mass spectroscopy data analysis

All data were analyzed and quantified with the MaxQuant software (version 1.6.3.4).[86] The false discovery rate was set to 1% for both proteins and peptides, and we specified a minimum peptide length of seven amino acids. The Andromeda search engine was used for the MS/MS spectra search against the *Rattus norvegius* database. Enzyme specificity was set as C-terminal to Arg and Lys, also allowing cleavage at proline bonds and a maximum of two missed cleavages. Dithiomethylation of cysteine was selected as fixed modification, and N-terminal protein acetylation and methionine oxidation as variable modifications. The "match between runs" feature of MaxQuant was used to transfer identifications to other liquid chromatography-tandem mass spectrometry runs based on their masses and retention time (maximum deviation 0.7 min), and this was also used in quantification experiments. Quantifications were performed with the label-free algorithm in MaxQuant.[86] Data analysis was performed using Perseus v.1.6.1.3 software[87] and R project v.3.6.0. Identifications mapping to more than one protein ID were discarded. Remaining identifications were mapped to gene names based on their ID using Uniprot database, and only uniquely mapping genes were kept for further analysis. Matrix was then further filtered to remove genes with less than 80% of positive values in one of the groups, and remaining missing data were imputed using QRILC method according to Wei et al.[88]

Integration of RNA-seq, small RNA-seq, and MS data

Differentially expressed miRNAs ($p_{adj} < 0.05$, log2FC > 1, and log2FC < −1) calculated by DESeq2 were used to predict mRNA targets in miRWalk database (http://mirwalk.umm.uni-heidelberg.de/). Log10 and DESeq2 normalized expression values of mRNA and log10 vst-transformed expression values of proteins without imputation, which were found among possible targets, were correlated using Pearson correlation with log10 and DESeq2 normalized expression values of miRNAs. The following were used correlation between miRNA and mRNA less than −0.85 and between miRNA and proteins less than 0. For visualization of interactom was used Cytoscape v.3.9.1.[81]

Cell cultures

All experiments used neural precursors differentiated from human-induced pluripotent (iPSC-NPs) cells derived from fetal lung fibroblast line (iMR 90; ATCC, Manassas, VA, USA). The cells were cultured in T75 flasks (Nunc) coated with poly-L-ornithine (P4957, Sigma-Aldrich) and laminin (L2020,Sigma-Aldrich) in a CO_2 incubator (MCO-170AICUVH-PE, Panasonic) at 37°C. Medium for NSC consisted of DMEM: F12 (21331-046) and neurobasal medium (21103049) (1: 1), supplements B27 (0080085SA) (1:50) and N2 (17502048) (1: 100), a mixture of penicillin and streptomycin antibiotics (15140122) (1: 200) (all from Gibco), epidermal growth factor (AF-100-15) (10 ng/mL), basic fibroblast growth factor (100-18B) (10 ng/mL), and brain derived growth factor (450-02) (20 ng/mL) (all from PeproTech); antibiotic primocin (ant-pm-05) (1: 500) (InvivoGen) was changed every other day.

To elucidate the role of the miR-20a-5p in the differentiation of neural stem cells, iPSC-NPs were seeded in 6 well plates coated with poly-L-ornithine and laminin with a working density about 60%–80% of confluence. After 24 h, the normal NSC medium was changed

to NSC differentiation medium containing 100 nM of miR-20a-5p inhibitor (339203 YCI0199790-FZC, QIAGEN) or 100 nM of negative control B (199007-121, QIAGEN) and cultured under these conditions for 72 h. Following this process, the miRNA inhibitor and scramble miRNA were omitted from medium, and differentiation continued with a medium consisting of DMEM: F12 and neurobasal medium (1: 1), supplements B27 (1:50) and N2 (1: 100), a mixture of penicillin and streptomycin antibiotics (1: 200) (all from Gibco), Shh (50 ng/mL) (100-45, PeproTech), retinoic acid (50nM) (Sigma-Aldrich), and primocin (1: 500) (InvivoGen). The cells were allowed to differentiate for 2 weeks, with half the volume of the medium being changed every other day. Following differentiation, the cells were washed with PBS and collected for further analysis.

To elucidate the role of the miR-20a-5p in the apoptotic processes, iPSC-NPs were seeded in 6 well plates coated with poly-L-ornithine and laminin with a working density about 60–80% of confluence. After 24 h, the normal NSC medium was changed to NSC medium containing 100 nM of H_2O_2 (Sigma-Aldrich) for 2 h. After creating the conditions of oxidative stress, cells were cultured with miR-20a-5p inhibitor or scumble miRNA both in concentration of 100nM during 72 h. Thus, several groups of samples were obtained: control (untreated) cells; cells treated with H_2O_2 only; cells treated with miR-20a inhibitor only; cells treated with H_2O_2 followed by miR-20a inhibitor and cells treated with H_2O_2 followed by scramble miRNA. Following the inhibition, cells were washed with PBS and harvested for further analysis.

OTS culture and *in vitro* lesion

OTSs were routinely extracted from 5-7-day old male Wistar rats. After anesthetizing them briefly with Vetbutal, animals were decapitated, and spine was isolated. To obtain the slices, the spine was placed on the Leica vibratome VT1200, alongside the blade, and was cut into 400μm slices and 1.5-2-cm-long slices. The slices were transferred onto the Millicell-CM (Millipore) membranes for further growth, three-four slices on each. The Millicell-CM membranes in 6-well plates were preequilibrated with 1mL of culture medium, consisted of 50% DMEM/F12, 10 mM HEPES, 25% HBSS, 25% horse serum (all from Thermo Scientific), 2 mmol/L L-glutamine (Sigma), 5 mg/mL glucose (BIOCEV), 1% amphotericine B (Sigma), and 0.4% penicillin-streptomycin (Thermo Scientific). OTSs were grown in humid conditions and 5% CO_2, at 34°C for 10 days. Lesions were made using surgical spring scissors, after which the medium was immediately replaced with either fresh medium (intact slices and lesioned control), medium containing 100 nM miR-20a-5p inhibitor, or medium containing 100 nM scrambled miRNA (negative control B). After 72 h, organotypic slices were harvested for Western blot or immunohistochemical staining.

Western blotting

The cells or organotypic tissue slices were homogenized on ice in RIPA lysis buffer (150 mM NaCl, 50 mM Tris (pH 8), 1% Triton X-100, 0.5% sodium deoxycholate, 0.1% sodium dodecyl sulfate) (all from Sigma-Aldrich) containing protease and phosphatase inhibitor (Thermo Scientific). The homogenized cells were incubated at 4°C for 40 min, then centrifuged at 14,000 RPM and 4°C for 20 min on a 5804 R centrifuge (Eppendorf). The resulting samples were frozen at −80°C. The Pierce TM BCA Protein Assay Kit (23225, Thermo Scientific) was used to determine the total protein concentration according to the manufacturer's instructions. Spectrophotometric measurements were performed using i-control software on an Infinite 200 PRO Multimode Reader (Tecan). Samples were immobilized using SDS sample buffer (80 mM Tris (pH 6.8), 2% SDS, 10% glycerol, 0.0006% bromophenol blue, 0.1 M DTT) (all from Sigma-Aldrich) for 5 min at 95°C. Electrophoresis was carried out for 20–30 min using Mini-PROTEAN TGXTM Precast Gels (4561084, Bio-Rad) with a gradient (8–14%) in electrophoresis buffer (25 mM Tris, 192 mM glycine, 0.1% SDS) (all from Sigma-Aldrich). Each well within the gel was loaded with 10 μg of total protein.

After electrophoresis, proteins were transferred from gels to PVDF membranes (Life Technologies) in transfer buffer (25 mM Tris, 192 mM glycine, pH 8.3; along with 20% methanol (v/v) (all from Sigma-Aldrich)) by Western Blot. For visualization of proteins, membranes were stained with Ponceau S Staining Solution (59803, Cell Signaling Technology). Tris-buffered saline/Tween 20 (TBST) buffer (20 mM Tris, 150 nM NaCl, 0.1% Tween 20; pH 7.5) (all from Sigma-Aldrich) was used to wash the membranes between steps.

In order to block nonspecific binding sites, the membranes were incubated with 5% milk solution (Cell Signaling Technology). For immunodetection, primary antibodies were prepared in 5% bovine serum albumin (BSA) solution (Cell Signaling Technology) or 5% milk solution (Cell Signaling Technology), or directly in TBST and incubated at 4°C overnight. Individual antibodies and their dilutions are described in Table S8. Subsequently, the membranes were washed in TBST and incubated with the appropriate secondary antibodies diluted in TBST for one hour on a shaker at RT. The membranes were then washed again with TBST solution. To visualize the staining, Super Signal West Dura kit (34076, Thermo Scientific) was used according to the manufacturer's instructions. Blots were captured on Azure c600 (Azure Biosystems) using Capture software. Image digitalization and analysis was performed using Fiji software. The results were normalized for endogenous control protein b-Actin (45kDa).

Immunohistochemical staining

For immunohistochemical staining, OTS were first fixed with 4% PFA (Penta, Czech Republic) and washed with phosphate buffered saline (PBS) (Thermo Fisher Scientific). Next, samples were permeabilized with 0.2% Triton (Sigma-Aldrich) in PBS for 30 min at RT in the dark. Nonspecific staining was blocked with 10% NGS (Sigma-Aldrich) serum solution in PBS for 30–45 min at RT in the dark. Primary antibodies diluted in 2% NGS and 0.1% Triton in PBS were added and incubated overnight at 4°C in the dark. Individual antibodies and their dilutions are described in Table S8. The next

day, the corresponding secondary antibodies were diluted 1:400 in the same solution that was used to dilute the primary antibodies and were used for immunodetection. Cell nuclei were visualized by adding DAPI (Sigma-Aldrich) solution prepared in PBS at a dilution of 1:2,500. OTS slides were removed from the wells and attached to microscopic coverslips using Aqua Poly/Mount (Polysciences) solution. Stained OTS were captured using a Carl Zeiss LSM 880 NLO confocal microscope (Carl Zeiss AG, Oberkochen, Germany).

DATA AND CODE AVAILABILITY

RNA-seq data generated in this study are available in NCBI's Gene Expression Omnibus and are accessible through GEO Series accession number GSE295958 (https://www.ncbi.nlm.nih.gov/geo/query/acc.cgi?acc=GSE295958). Other data are available from the corresponding authors upon reasonable request.

ACKNOWLEDGMENTS

We thank the Laboratory of Mass Spectrometry, Biocev, Charles University, Faculty of Science, for performing LC-MS analysis and GeneCore facility, Biocev, Institute of Biotechnology, for assisting with RNA-seq analysis. This study was supported by the Czech Science Foundation (23-05327S and 23-06269S); Czech Health Research Council (NU21-08-00286); MULTIOMICS_CZ (CZ.02.01.01/00/23_020/0008540); Ministry of Education, Youth and Sport – CZ.02.01.01/00/22_008/0004562; and institutional support RVO 86652036. All the experiments were performed in accordance with the European Communities Council Directive of 22nd of September 2010 (2010/63/EU) regarding the use of animals in research and were approved by the Ethics Committee of the Institute of Experimental Medicine CAS and subsequently by the Section Committee of Czech Academy of Sciences, Prague, Czech Republic (project no. 54/2017, approved 14th of July 2017).

AUTHOR CONTRIBUTIONS

R.A.K., data curation, formal analysis, investigation, methodology, validation, visualization, writing – original draft, and writing – review and editing; S.C., data curation and formal analysis; I.A., data curation and formal analysis; D.Z., data curation and formal analysis; E.R., data curation and formal analysis; P. Androvic, data curation; P. Abaffy, data curation and formal analysis; L.U.-M., data curation and formal analysis; M.K., funding acquisition; N.R., conceptualization, funding acquisition, supervision, and writing – review and editing; L.V., conceptualization, funding acquisition, supervision, and writing – review and editing.

DECLARATION OF INTERESTS

The authors declare no competing interests.

SUPPLEMENTAL INFORMATION

Supplemental information can be found online at https://doi.org/10.1016/j.omtn.2025.102746.

REFERENCES

1. Ding, W., Hu, S., Wang, P., Kang, H., Peng, R., Dong, Y., and Li, F. (2022). Spinal Cord Injury: The Global Incidence, Prevalence, and Disability From the Global Burden of Disease Study 2019. Spine *47*, 1532–1540. https://doi.org/10.1097/BRS.0000000000004417.

2. Merritt, C.H., Taylor, M.A., Yelton, C.J., and Ray, S.K. (2019). Economic Impact of Traumatic Spinal Cord Injuries in the United States. Neuroimmunol. Neuroinflamm. *6*, 9. https://doi.org/10.20517/2347-8659.2019.15.

3. Ramer, L.M., Ramer, M.S., and Bradbury, E.J. (2014). Restoring Function after Spinal Cord Injury: Towards Clinical Translation of Experimental Strategies. Lancet Neurol. *13*, 1241–1256. https://doi.org/10.1016/S1474-4422(14)70144-9.

4. Carmel, J.B., Galante, A., Soteropoulos, P., Tolias, P., Recce, M., Young, W., and Hart, R.P. (2001). Gene Expression Profiling of Acute Spinal Cord Injury Reveals Spreading Inflammatory Signals and Neuron Loss. Physiol. Genomics *7*, 201–213. https://doi.org/10.1152/physiolgenomics.00074.2001.

5. Chamankhah, M., Eftekharpour, E., Karimi-Abdolrezaee, S., Boutros, P.C., San-Marina, S., and Fehlings, M.G. (2013). Genome-Wide Gene Expression Profiling of Stress Response in a Spinal Cord Clip Compression Injury Model. BMC Genom. *14*, 583. https://doi.org/10.1186/1471-2164-14-583.

6. Chen, K., Deng, S., Lu, H., Zheng, Y., Yang, G., Kim, D., Cao, Q., and Wu, J.Q. (2013). RNA-Seq Characterization of Spinal Cord Injury Transcriptome in Acute/Subacute Phases: A Resource for Understanding the Pathology at the Systems Level. PLoS One *8*, 72567. https://doi.org/10.1371/journal.pone.0072567.

7. De Biase, A., Knoblach, S.M., Di Giovanni, S., Fan, C., Molon, A., Hoffman, E.P., and Faden, A.I. (2005). Gene Expression Profiling of Experimental Traumatic Spinal Cord Injury as a Function of Distance from Impact Site and Injury Severity. Physiol. Genomics *22*, 368–381. https://doi.org/10.1152/physiolgenomics.00081.2005.

8. Milich, L.M., Choi, J.S., Ryan, C., Cerqueira, S.R., Benavides, S., Yahn, S.L., Tsoulfas, P., and Lee, J.K. (2021). Single-Cell Analysis of the Cellular Heterogeneity and Interactions in the Injured Mouse Spinal Cord. J. Exp. Med. *218*, e20210040–e20212504. https://doi.org/10.1084/jem.20210040.

9. Matson, K.J.E., Russ, D.E., Kathe, C., Hua, I., Maric, D., Ding, Y., Krynitsky, J., Pursley, R., Sathyamurthy, A., Squair, J.W., et al. (2022). Single Cell Atlas of Spinal Cord Injury in Mice Reveals a Pro-Regenerative Signature in Spinocerebellar Neurons. Nat. Commun. *13*, 5628. https://doi.org/10.1038/s41467-022-33184-1.

10. Matson, K.J.E., Russ, D.E., Kathe, C., Maric, D., Hua, I., Krynitsky, J., Pursley, R., Sathyamurthy, A., Squair, J.W., Courtine, G., and Levine, A.J. (2021). A Single Cell Atlas of Spared Tissue Below a Spinal Cord Injury Reveals Cellular Mechanisms of Repair. Preprint at bioRxiv. https://doi.org/10.1101/2021.04.28.441862.

11. Chakraborty, C., Sharma, A.R., Sharma, G., and Lee, S.S. (2021). Therapeutic Advances of MiRNAs: A Preclinical and Clinical Update. J. Adv. Res. *28*, 127–138. https://doi.org/10.1016/J.JARE.2020.08.012.

12. Rupaimoole, R., and Slack, F.J. (2017). MicroRNA Therapeutics: Towards a New Era for the Management of Cancer and Other Diseases. Nat. Rev. Drug Discov. *16*, 203–222. https://doi.org/10.1038/nrd.2016.246.

13. Kim, T., and Croce, C.M. (2023). MicroRNA: Trends in Clinical Trials of Cancer Diagnosis and Therapy Strategies. Exp. Mol. Med. *55*, 1314–1321. https://doi.org/10.1038/s12276-023-01050-9.

14. Wang, W., Li, J., Zhang, Z., Ma, H., Li, Q., Yang, H., Li, M., and Liu, L. (2021). Genome-Wide Analysis of Acute Traumatic Spinal Cord Injury-Related RNA Expression Profiles and Uncovering of a Regulatory Axis in Spinal Fibrotic Scars. Cell Prolif. *54*, e12951-15. https://doi.org/10.1111/cpr.12951.

15. Peng, P., Zhang, B., Huang, J., Xing, C., Liu, W., Sun, C., Guo, W., Yao, S., Ruan, W., Ning, G., et al. (2020). Identification of a CircRNA-MiRNA-MRNA Network to Explore the Effects of CircRNAs on Pathogenesis and Treatment of Spinal Cord Injury. Life Sci. *257*, 118039. https://doi.org/10.1016/j.lfs.2020.118039.

16. Basso, D.M., Beattie, M.S., and Bresnahan, J.C. (1995). A Sensitive and Reliable Locomotor Rating Scale for Open Field Testing in Rats. J. Neurotrauma *12*, 1–21. https://doi.org/10.1089/neu.1995.12.1.

17. Hunter, M., Spiller, K.J., Dominique, M.A., Xu, H., Hunter, F.W., Fang, T.C., Canter, R.G., Roberts, C.J., Ransohoff, R.M., Trojanowski, J.Q., and Lee, V.M.Y. (2021). Microglial Transcriptome Analysis in the RNLS8 Mouse Model of TDP-43 Proteinopathy Reveals Discrete Expression Profiles Associated with Neurodegenerative Progression and Recovery. Acta Neuropathol. Commun. *9*, 140. https://doi.org/10.1186/s40478-021-01239-x.

18. Shin, D., Howng, S.Y.B., Ptáček, L.J., and Fu, Y.H. (2012). MiR-32 and Its Target SLC45A3 Regulate the Lipid Metabolism of Oligodendrocytes and Myelin. Neuroscience *213*, 29–37. https://doi.org/10.1016/j.neuroscience.2012.03.054.

19. Reimand, J., Isserlin, R., Voisin, V., Kucera, M., Tannus-Lopes, C., Rostamianfar, A., Wadi, L., Meyer, M., Wong, J., Xu, C., et al. (2019). Pathway Enrichment Analysis and Visualization of Omics Data Using g:Profiler, GSEA, Cytoscape and EnrichmentMap. Nat. Protoc. *14*, 482–517. https://doi.org/10.1038/s41596-018-0103-9.

20. Yuan, H., Zhang, B., Ma, J., Zhang, Y., Tuo, Y., and Li, X. (2022). Analysis of Gene Expression Profiles in Two Spinal Cord Injury Models. Eur. J. Med. Res. *27*, 156. https://doi.org/10.1186/S40001-022-00785-X/TABLES/2.

21. Li, Y., Chen, Y., Li, X., Wu, J., Pan, J.Y., Cai, R.X., Yang, R.Y., and Wang, X.D. (2019). RNA Sequencing Screening of Differentially Expressed Genes after Spinal Cord Injury. Neural Regen. Res. *14*, 1583–1593. https://doi.org/10.4103/1673-5374.255994.

22. Shi, L.L., Zhang, N., Xie, X.M., Chen, Y.J., Wang, R., Shen, L., Zhou, J.S., Hu, J.G., and Lü, H.Z. (2017). Transcriptome Profile of Rat Genes in Injured Spinal Cord at Different Stages by RNA-Sequencing. BMC Genom. *18*, 173. https://doi.org/10.1186/s12864-017-3532-x.

23. Squair, J.W., Tigchelaar, S., Moon, K.M., Liu, J., Tetzlaff, W., Kwon, B.K., Krassioukov, A.V., West, C.R., Foster, L.J., and Skinnider, M.A. (2018). Integrated Systems Analysis Reveals Conserved Gene Networks Underlying Response to Spinal Cord Injury. eLife *7*, e39188-23. https://doi.org/10.7554/eLife.39188.

24. Newman, A.M., Steen, C.B., Liu, C.L., Gentles, A.J., Chaudhuri, A.A., Scherer, F., Khodadoust, M.S., Esfahani, M.S., Luca, B.A., Steiner, D., et al. (2019). Determining Cell Type Abundance and Expression from Bulk Tissues with Digital Cytometry. Nat. Biotechnol. *37*, 773–782. https://doi.org/10.1038/s41587-019-0114-2.

25. Franzén, O., Gan, L.M., and Björkegren, J.L.M. (2019). PanglaoDB: A Web Server for Exploration of Mouse and Human Single-Cell RNA Sequencing Data. Database *2019*, 46. https://doi.org/10.1093/database/baz046.

26. Langfelder, P., and Horvath, S. (2008). WGCNA: An R Package for Weighted Correlation Network Analysis. BMC Bioinf. *9*, 559. https://doi.org/10.1186/1471-2105-9-559.

27. Barberán-Soler, S., Vo, J.M., Hogans, R.E., Dallas, A., Johnston, B.H., and Kazakov, S.A. (2018). Decreasing MiRNA Sequencing Bias Using a Single Adapter and Circularization Approach. Genome Biol. *19*, 1–9. https://doi.org/10.1186/S13059-018-1488-Z/FIGURES/5.

28. Sticht, C., De La Torre, C., Parveen, A., and Gretz, N. (2018). MiRWalk: An Online Resource for Prediction of MicroRNA Binding Sites. PLoS One *13*, e0206239. https://doi.org/10.1371/JOURNAL.PONE.0206239.

29. Plotnikov, A., Zehorai, E., Procaccia, S., and Seger, R. (2011). The MAPK Cascades: Signaling Components, Nuclear Roles and Mechanisms of Nuclear Translocation. Biochim. Biophys. Acta *1813*, 1619–1633. https://doi.org/10.1016/J.BBAMCR.2010.12.012.

30. Zhou, Y., Wang, Z., Li, J., Li, X., and Xiao, J. (2018). Fibroblast Growth Factors in the Management of Spinal Cord Injury. J. Cell Mol. Med. *22*, 25–37. https://doi.org/10.1111/jcmm.13353.

31. Ponomarev, E.D., Veremeyko, T., Barteneva, N., Krichevsky, A.M., and Weiner, H.L. (2011). MicroRNA-124 Promotes Microglia Quiescence and Suppresses EAE by Deactivating Macrophages via the C/EBP-α-PU.1 Pathway. Nat. Med. *17*, 64–70. https://doi.org/10.1038/nm.2266.

32. Zhang, C., Talifu, Z., Xu, X., Liu, W., Ke, H., Pan, Y., Li, Y., Bai, F., Jing, Y., Li, Z., et al. (2023). MicroRNAs in Spinal Cord Injury: A Narrative Review. Front. Mol. Neurosci. *16*, 1099256. https://doi.org/10.3389/FNMOL.2023.1099256/BIBTEX.

33. Wei, J., Wang, J., Zhou, Y., Yan, S., Li, K., and Lin, H. (2016). MicroRNA-146a Contributes to SCI Recovery via Regulating TRAF6 and IRAK1 Expression. BioMed Res. Int. *2016*, 4013487. https://doi.org/10.1155/2016/4013487.

34. Yang, P., Cai, L., Zhang, G., Bian, Z., and Han, G. (2017). The Role of the MiR-17–92 Cluster in Neurogenesis and Angiogenesis in the Central Nervous System of Adults. J. Neurosci. Res. *95*, 1574–1581. https://doi.org/10.1002/JNR.23991.

35. Pan, W.L., Chopp, M., Fan, B., Zhang, R., Wang, X., Hu, J., Zhang, X.M., Zhang, Z.G., and Liu, X.S. (2019). Ablation of the MicroRNA-17-92 Cluster in Neural Stem Cells Diminishes Adult Hippocampal Neurogenesis and Cognitive Function. FASEB J. *33*, 5257–5267. https://doi.org/10.1096/FJ.201801019R.

36. Amemori, T., Romanyuk, N., Jendelova, P., Herynek, V., Turnovcova, K., Prochazka, P., Kapcalova, M., Cocks, G., Price, J., and Sykova, E. (2013). Human Conditionally Immortalized Neural Stem Cells Improve Locomotor Function after Spinal Cord Injury in the Rat. Stem Cell Res. Ther. *4*, 68. https://doi.org/10.1186/SCRT219/FIGURES/7.

37. Ruzicka, J., Machova-Urdzikova, L., Gillick, J., Amemori, T., Romanyuk, N., Karova, K., Zaviskova, K., Dubisova, J., Kubinova, S., Murali, R., et al. (2017). A Comparative Study of Three Different Types of Stem Cells for Treatment of Rat Spinal Cord Injury. Cell Transplant. *26*, 585–603. https://doi.org/10.3727/096368916X693671.

38. Romanyuk, N., Amemori, T., Turnovcova, K., Prochazka, P., Onteniente, B., Sykova, E., and Jendelova, P. (2015). Beneficial Effect of Human Induced Pluripotent Stem Cell-Derived Neural Precursors in Spinal Cord Injury Repair. Cell Transplant. *24*, 1781–1797. https://doi.org/10.3727/096368914X684042.

39. Ruzicka, J., Romanyuk, N., Jirakova, K., Hejcl, A., Janouskova, O., Machova, L.U., Bochin, M., Pradny, M., Vargova, L., and Jendelova, P. (2019). The Effect of IPS-Derived Neural Progenitors Seeded on Laminin-Coated PHEMA-MOETACl Hydrogel with Dual Porosity in a Rat Model of Chronic Spinal Cord Injury. Cell Transplant. *28*, 400–412. https://doi.org/10.1177/0963689718823705.

40. Amemori, T., Ruzicka, J., Romanyuk, N., Jhanwar-Uniyal, M., Sykova, E., and Jendelova, P. (2015). Comparison of Intraspinal and Intrathecal Implantation of Induced Pluripotent Stem Cell-Derived Neural Precursors for the Treatment of Spinal Cord Injury in Rats. Stem Cell Res. Ther. *6*, 257. https://doi.org/10.1186/S13287-015-0255-2/FIGURES/4.

41. Ruzicka, J., Romanyuk, N., Hejcl, A., Vetrik, M., Hruby, M., Cocks, G., Cihlar, J., Pradny, M., Price, J., Sykova, E., et al. (2013). Treating Spinal Cord Injury in Rats with a Combination of Human Fetal Neural Stem Cells and Hydrogels Modified with Serotonin. Acta Neurobiol. Exp. *73*, 102–115. https://doi.org/10.55782/ane-2013-1925.

42. Wertz, M.H., Winden, K., Neveu, P., Ng, S.Y., Ercan, E., and Sahin, M. (2016). Cell-Type-Specific MiR-431 Dysregulation in a Motor Neuron Model of Spinal Muscular Atrophy. Hum. Mol. Genet. *25*, 2168–2181. https://doi.org/10.1093/HMG/DDW084.

43. Niu, X., Zhu, H.L., Liu, Q., Yan, J.F., and Li, M.L. (2021). MiR-194-5p Serves as a Potential Biomarker and Regulates the Proliferation and Apoptosis of Hippocampus Neuron in Children with Temporal Lobe Epilepsy. J. Chin. Med. Assoc. *84*, 510–516. https://doi.org/10.1097/JCMA.0000000000000518.

44. Konrad, K.D., and Song, J.L. (2023). MicroRNA-124 Regulates Notch and NeuroD1 to Mediate Transition States of Neuronal Development. Dev. Neurobiol. *83*, 3–27. https://doi.org/10.1002/DNEU.22902.

45. Ziu, M., Fletcher, L., Rana, S., Jimenez, D.F., and Digicaylioglu, M. (2011). Temporal Differences in MicroRNA Expression Patterns in Astrocytes and Neurons after Ischemic Injury. PLoS One *6*, e14724. https://doi.org/10.1371/JOURNAL.PONE.0014724.

46. Xu, D., and Guo, Q. (2024). MiR-26a Improves Microglial Activation and Neuronal Apoptosis in a Rat Model of Cerebral Infarction by Regulating the TREM1-TLR4/MyD88/NF-KB Axis. Dev. Neurosci. *46*, 221–236. https://doi.org/10.1159/000533813.

47. Ma, Q., Zhao, H., Tao, Z., Wang, R., Liu, P., Han, Z., Ma, S., Luo, Y., and Jia, J. (2016). MicroRNA-181c Exacerbates Brain Injury in Acute Ischemic Stroke. Aging Dis. *7*, 705–714. https://doi.org/10.14336/AD.2016.0320.

48. Jiao, P., Wang, X.P., Luoreng, Z.M., Yang, J., Jia, L., Ma, Y., and Wei, D.W. (2021). Mir-223: An Effective Regulator of Immune Cell Differentiation and Inflammation. Int. J. Biol. Sci. *17*, 2308–2322. https://doi.org/10.7150/ijbs.59876.

49. Tigchelaar, S., Streijger, F., Sinha, S., Flibotte, S., Manouchehri, N., So, K., Shortt, K., Okon, E., Rizzuto, M.A., Malenica, I., et al. (2017). Serum MicroRNAs Reflect Injury Severity in a Large Animal Model of Thoracic Spinal Cord Injury. Sci. Rep. *7*, 1376. https://doi.org/10.1038/s41598-017-01299-x.

50. Leem, K.H., Kim, S., Kim, H.W., and Park, H.J. (2023). Downregulation of MicroRNA-330-5p Induces Manic-like Behaviors in REM Sleep-deprived Rats by Enhancing Tyrosine Hydroxylase Expression. CNS Neurosci. Ther. *29*, 1525–1536. https://doi.org/10.1111/CNS.14121.

51. Madathil, S.K., Nelson, P.T., Saatman, K.E., and Wilfred, B.R. (2011). MicroRNAs in CNS Injury: Potential Roles and Therapeutic Implications. Bioessays *33*, 21–26. https://doi.org/10.1002/BIES.201000069.

52. Kamal, M.A., Mushtaq, G., and Greig, N.H. (2015). Current Update on Synopsis of MiRNA Dysregulation in Neurological Disorders. CNS Neurol. Disord.: Drug Targets *14*, 492–501. https://doi.org/10.2174/1871527314666150225143637.

53. Liu, N.K., Wang, X.F., Lu, Q.B., and Xu, X.M. (2009). Altered MicroRNA Expression Following Traumatic Spinal Cord Injury. Exp. Neurol. *219*, 424–429. https://doi.org/10.1016/j.expneurol.2009.06.015.

54. Strickland, E.R., Hook, M.A., Balaraman, S., Huie, J.R., Grau, J.W., and Miranda, R.C. (2011). MicroRNA Dysregulation Following Spinal Cord Contusion: Implications for Neural Plasticity and Repair. Neuroscience *186*, 146–160. https://doi.org/10.1016/j.neuroscience.2011.03.063.

55. Yunta, M., Nieto-Díaz, M., Esteban, F.J., Caballero-López, M., Navarro-Ruíz, R., Reigada, D., Pita-Thomas, D.W., del Águila, A., Muñoz-Galdeano, T., and Maza, R.M. (2012). MicroRNA Dysregulation in the Spinal Cord Following Traumatic Injury. PLoS One *7*, e34534. https://doi.org/10.1371/journal.pone.0034534.

56. Jee, M.K., Jung, J.S., Im, Y.B., Jung, S.J., and Kang, S.K. (2012). Silencing of MiR20a Is Crucial for Ngn1-Mediated Neuroprotection in Injured Spinal Cord. Hum. Gene Ther. *23*, 508–520. https://doi.org/10.1089/hum.2011.121.

57. Gao, Z., Ure, K., Ables, J.L., Lagace, D.C., Nave, K.A., Goebbels, S., Eisch, A.J., and Hsieh, J. (2009). Neurod1 Is Essential for the Survival and Maturation of Adult-Born Neurons. Nat. Neurosci. *12*, 1090–1092. https://doi.org/10.1038/nn.2385.

58. Puls, B., Ding, Y., Zhang, F., Pan, M., Lei, Z., Pei, Z., Jiang, M., Bai, Y., Forsyth, C., Metzger, M., et al. (2020). Regeneration of Functional Neurons After Spinal Cord Injury via in Situ NeuroD1-Mediated Astrocyte-to-Neuron Conversion. Front. Cell Dev. Biol. *8*, 591883. https://doi.org/10.3389/FCELL.2020.591883/BIBTEX.

59. Hunt, D., Coffin, R.S., Prinjha, R.K., Campbell, G., and Anderson, P.N. (2003). Nogo-A Expression in the Intact and Injured Nervous System. Mol. Cell. Neurosci. *24*, 1083–1102. https://doi.org/10.1016/j.mcn.2003.09.002.

60. Schweigreiter, R., and Bandtlow, C.E. (2006). Nogo in the Injured Spinal Cord. J. Neurotrauma *23*, 384–396. https://doi.org/10.1089/NEU.2006.23.384.

61. Cafferty, W.B.J., Duffy, P., Huebner, E., and Strittmatter, S.M. (2010). MAG and OMgp Synergize with Nogo-A to Restrict Axonal Growth and Neurological Recovery after Spinal Cord Trauma. J. Neurosci. *30*, 6825–6837. https://doi.org/10.1523/JNEUROSCI.6239-09.2010.

62. Cheng, X., Yu, Z., Xu, J., Quan, D., and Long, H. (2021). Pathophysiological Changes and the Role of Notch-1 Activation After Decompression in a Compressive Spinal Cord Injury Rat Model. Front. Neurosci. *15*, 579431. https://doi.org/10.3389/FNINS.2021.579431/BIBTEX.

63. Tan, Z., Qin, S., Yuan, Y., Hu, X., Huang, X., Liu, H., Pu, Y., He, C., and Su, Z. (2022). NOTCH1 Signaling Regulates the Latent Neurogenic Program in Adult Reactive Astrocytes after Spinal Cord Injury. Theranostics *12*, 4548–4563. https://doi.org/10.7150/THNO.71378.

64. Peng, Z., Li, X., Fu, M., Zhu, K., Long, L., Zhao, X., Chen, Q., Deng, D.Y.B., and Wan, Y. (2019). Inhibition of Notch1 Signaling Promotes Neuronal Differentiation and Improves Functional Recovery in Spinal Cord Injury through Suppressing the Activation of Ras Homolog Family Member A. J. Neurochem. *150*, 709–722. https://doi.org/10.1111/JNC.14833.

65. Kakinohana, M., Kida, K., Minamishima, S., Atochin, D.N., Huang, P.L., Kaneki, M., and Ichinose, F. (2011). Delayed Paraplegia after Spinal Cord Ischemic Injury Requires Caspase-3 Activation in Mice. Stroke *42*, 2302–2307. https://doi.org/10.1161/STROKEAHA.110.600429.

66. Citron, B.A., Arnold, P.M., Sebastian, C., Qin, F., Malladi, S., Ameenuddin, S., Landis, M.E., and Festoff, B.W. (2000). Rapid Upregulation of Caspase-3 in Rat Spinal Cord after Injury: MRNA, Protein, and Cellular Localization Correlates with Apoptotic Cell Death. Exp. Neurol. *166*, 213–226. https://doi.org/10.1006/exnr.2000.7523.

67. DeGiosio, R.A., Grubisha, M.J., MacDonald, M.L., McKinney, B.C., Camacho, C.J., and Sweet, R.A. (2022). More than a Marker: Potential Pathogenic Functions of MAP2. Front. Mol. Neurosci. *15*, 974890. https://doi.org/10.3389/FNMOL.2022.974890/XML.

68. Papasozomenos, S.C., Binder, L.I., Bender, P.K., and Payne, M.R. (1985). Microtubule-Associated Protein 2 within Axons of Spinal Motor Neurons: Associations with Microtubules and Neurofilaments in Normal and β,β'-Iminodipropionitrile-Treated Axons. J. Cell Biol. *100*, 74–85. https://doi.org/10.1083/JCB.100.1.74.

69. Urdzikova, L., Urdzikova, U., and Vanicky, I. (2006). Post-Traumatic Moderate Systemic Hyperthermia Worsens Behavioural Outcome after Spinal Cord Injury in the Rat. Spinal Cord *44*, 113–119. https://doi.org/10.1038/sj.sc.3101792.

70. Ferrier, J., Marchand, F., and Balayssac, D. (2016). Assessment of Mechanical Allodynia in Rats Using the Electronic Von Frey Test. Bio. Protoc. *6*, e1933. https://doi.org/10.21769/BIOPROTOC.1933.

71. Martinov, T., Mack, M., Sykes, A., and Chatterjea, D. (2013). Measuring Changes in Tactile Sensitivity in the Hind Paw of Mice Using an Electronic von Frey Apparatus. J. Vis. Exp. *2013*, e51212. https://doi.org/10.3791/51212.

72. Bolger, A.M., Lohse, M., and Usadel, B. (2014). Trimmomatic: A Flexible Trimmer for Illumina Sequence Data. Bioinformatics *30*, 2114–2120. https://doi.org/10.1093/bioinformatics/btu170.

73. Kopylova, E., Noé, L., and Touzet, H. (2012). SortMeRNA: Fast and Accurate Filtering of Ribosomal RNAs in Metatranscriptomic Data. Bioinformatics *28*, 3211–3217. https://doi.org/10.1093/bioinformatics/bts611.

74. Dobin, A., Davis, C.A., Schlesinger, F., Drenkow, J., Zaleski, C., Jha, S., Batut, P., Chaisson, M., and Gingeras, T.R. (2013). STAR: Ultrafast Universal RNA-Seq Aligner. Bioinformatics *29*, 15–21. https://doi.org/10.1093/bioinformatics/bts635.

75. Anders, S., Pyl, P.T., and Huber, W. (2015). HTSeq-A Python Framework to Work with High-Throughput Sequencing Data. Bioinformatics *31*, 166–169. https://doi.org/10.1093/bioinformatics/btu638.

76. Martin, M. (2011). Cutadapt Removes Adapter Sequences from High-Throughput Sequencing Reads. EMBnet. J. *17*, 10. https://doi.org/10.14806/ej.17.1.200.

77. Langmead, B., Trapnell, C., Pop, M., and Salzberg, S.L. (2009). Ultrafast and Memory-Efficient Alignment of Short DNA Sequences to the Human Genome. Genome Biol. *10*, R25. https://doi.org/10.1186/GB-2009-10-3-R25/TABLES/5.

78. Kozomara, A., Birgaoanu, M., and Griffiths-Jones, S. (2019). MiRBase: From MicroRNA Sequences to Function. Nucleic Acids Res. *47*, D155–D162. https://doi.org/10.1093/nar/gky1141.

79. Love, M.I., Huber, W., and Anders, S. (2014). Moderated Estimation of Fold Change and Dispersion for RNA-Seq Data with DESeq2. Genome Biol. *15*, 550. https://doi.org/10.1186/s13059-014-0550-8.

80. Subramanian, A., Tamayo, P., Mootha, V.K., Mukherjee, S., Ebert, B.L., Gillette, M.A., Paulovich, A., Pomeroy, S.L., Golub, T.R., Lander, E.S., and Mesirov, J.P. (2005). Gene Set Enrichment Analysis: A Knowledge-Based Approach for Interpreting Genome-Wide Expression Profiles. Proc. Natl. Acad. Sci. USA *102*, 15545–15550. https://doi.org/10.1073/PNAS.0506580102.

81. Shannon, P., Markiel, A., Ozier, O., Baliga, N.S., Wang, J.T., Ramage, D., Amin, N., Schwikowski, B., and Ideker, T. (2003). A Software Environment for Integrated Models of Biomolecular Interaction Networks. Genome Res. *13*, 2498–2504. https://doi.org/10.1101/GR.1239303.

82. Hao, Y., Hao, S., Andersen-Nissen, E., Mauck, W.M., Zheng, S., Butler, A., Lee, M.J., Wilk, A.J., Darby, C., Zager, M., et al. (2021). Integrated Analysis of Multimodal Single-Cell Data. Cell *184*, 3573–3587.e29. https://doi.org/10.1016/j.cell.2021.04.048.

83. Hughes, C.S., Moggridge, S., Müller, T., Sorensen, P.H., Morin, G.B., and Krijgsveld, J. (2018). Single-Pot, Solid-Phase-Enhanced Sample Preparation for Proteomics Experiments. Nat. Protoc. *14*, 68–85. https://doi.org/10.1038/s41596-018-0082-x.

84. Rappsilber, J., Mann, M., and Ishihama, Y. (2007). Protocol for Micro-Purification, Enrichment, Pre-Fractionation and Storage of Peptides for Proteomics Using StageTips. Nat. Protoc. *2*, 1896–1906. https://doi.org/10.1038/NPROT.2007.261.

85. Hebert, A.S., Richards, A.L., Bailey, D.J., Ulbrich, A., Coughlin, E.E., Westphall, M.S., and Coon, J.J. (2014). The One Hour Yeast Proteome. Mol. Cell. Proteomics *13*, 339–347. https://doi.org/10.1074/MCP.M113.034769.

86. Cox, J., and Mann, M. (2008). MaxQuant Enables High Peptide Identification Rates, Individualized p.p.b.-Range Mass Accuracies and Proteome-Wide Protein Quantification. Nat. Biotechnol. *26*, 1367–1372. https://doi.org/10.1038/nbt.1511.

87. Tyanova, S., Temu, T., Sinitcyn, P., Carlson, A., Hein, M.Y., Geiger, T., Mann, M., and Cox, J. (2016). The Perseus Computational Platform for Comprehensive Analysis of (Prote)Omics Data. Nat. Methods *13*, 731–740. https://doi.org/10.1038/nmeth.3901.

88. Wei, R., Wang, J., Su, M., Jia, E., Chen, S., Chen, T., and Ni, Y. (2018). Missing Value Imputation Approach for Mass Spectrometry-Based Metabolomics Data. Sci. Rep. *8*, 663. https://doi.org/10.1038/s41598-017-19120-0.

OMTN, Volume *36*

Supplemental information

Integrated multi-omics profiling uncovers miRNA-guided regulatory networks after spinal cord injury in rats

Ruslan A. Klassen, Sarka Chytilova, Ivan Arzhanov, Daniel Zucha, Eva Rohlova, Peter Androvic, Pavel Abaffy, Lucia Urdzikova-Machova, Mikael Kubista, Nataliya Romanyuk, and Lukas Valihrach

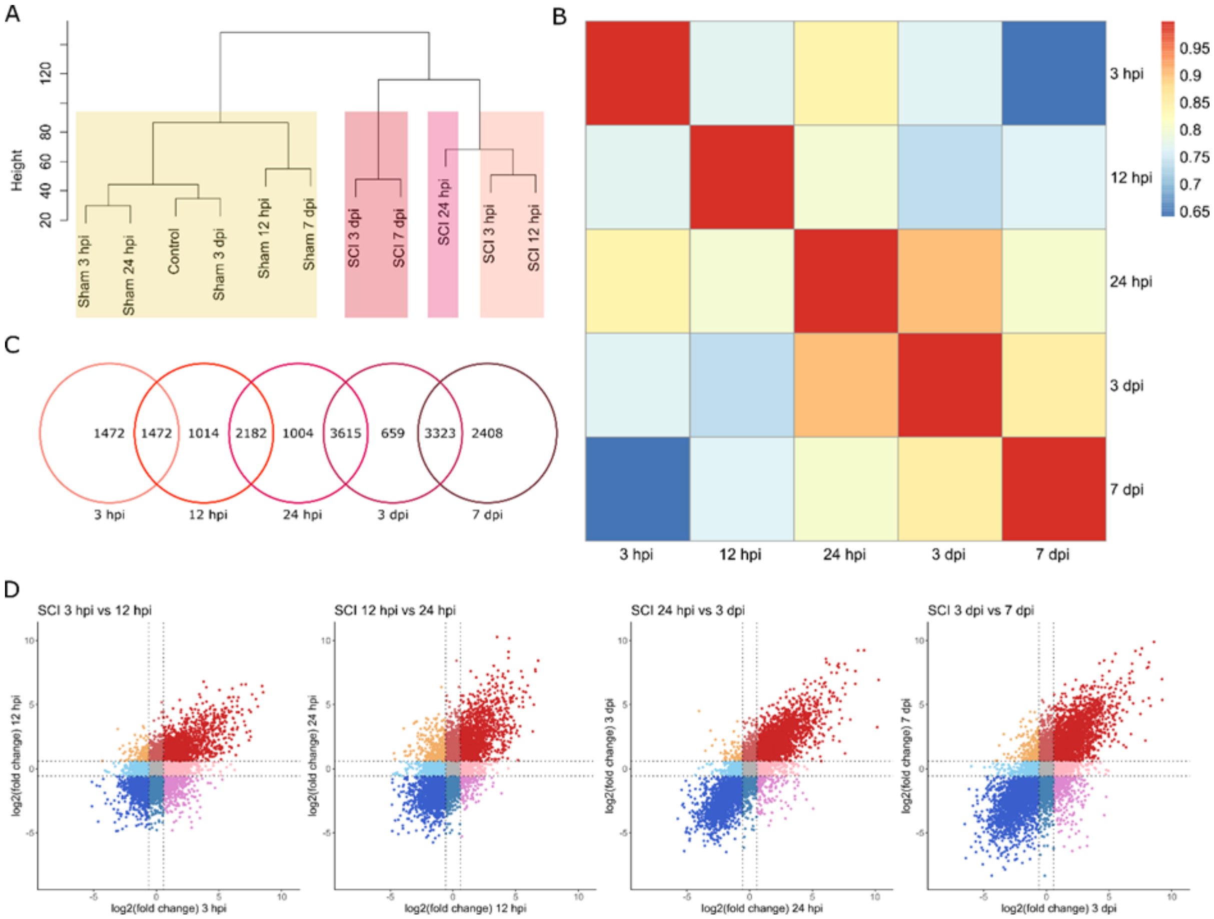

Figure S1: Sample similarity analysis.

A. Hierarchical clustering dendrogram of sample groups.

B. Pearson correlations of all DEGs between individual time points.

C. Overlap of DEGs between time points in ascending order from 3 hpi to 7 dpi ($p_{adj} < 0.05$ and $|\log_2 FC| > 0.58$).

D. Scatterplot comparing $\log_2FC$ of DEGs between consecutive time points. Genes with $\log_2FC > 0.58$ are highlighted in red, genes with $\log_2FC < -0.58$ are highlighted in blue, and genes with $\log_2FC$ between -0.58 and 0.58 in grey. Genes that switch from up- to downregulated are highlighted in violet, and from down- to upregulated in orange.

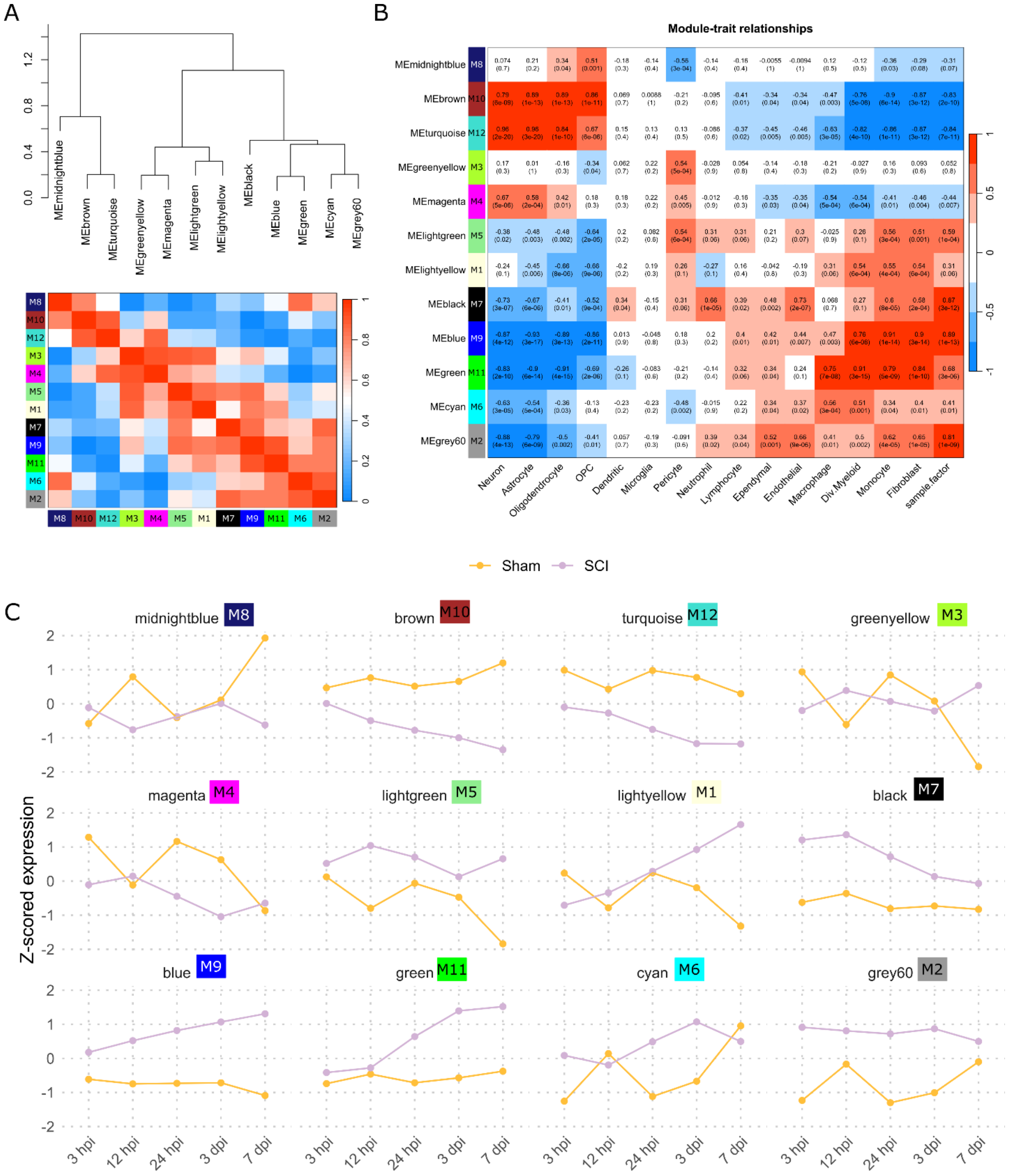

Figure S2: WGCNA module eigengene characterization.

A. Visualization of the eigengene network representing relationships among the modules. Hierarchical clustering dendrogram of modules eigengenes. The heatmap shows module eigengene adjacency.

B. Module-trait correlation analysis. Heatmap showing correlations between WGCNA module eigengenes and deconvoluted cell-specific profiles (see Fig. 4B) as well as injury status (sample.factor). Pearson correlation coefficients with p-values in brackets are displayed.

C. Mean expression profiles of all WGCNA modules.

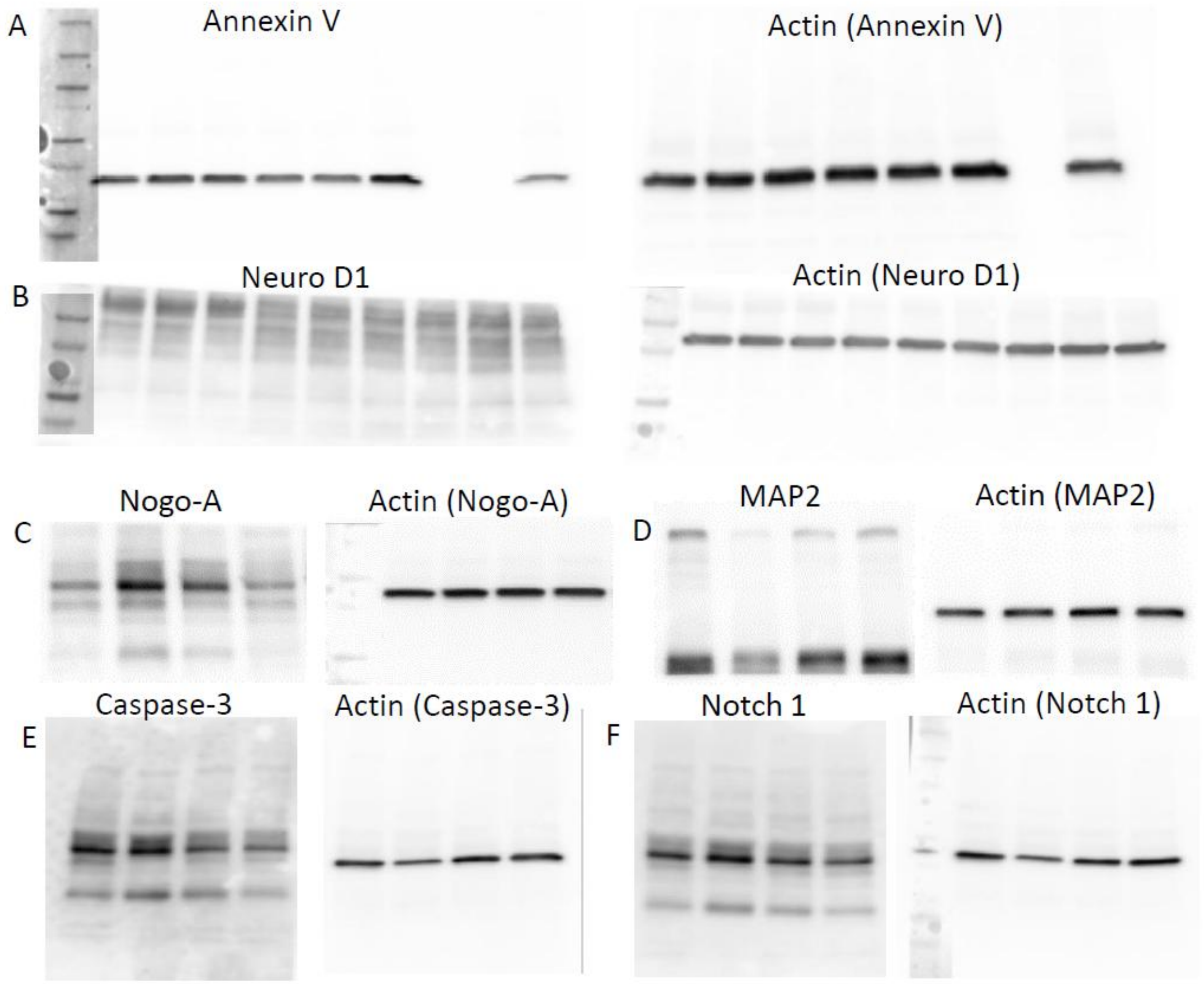

Figure S3: Raw images of blots presented in Figure 8.

Representative raw images of Western blots used to analyze levels of neuro-specific and apoptotic proteins shown in Figure 8. Actin was used as an endogenous control in each pair of images. Proteins analyzed: (A) Annexin V, (B) NeuroD1, (C) Nogo-A, (D) MAP2, (E) Caspase-3, and (F) Notch1. Nogo-A – neurite outgrowth inhibitor-A, MAP2 – microtubule-associated protein 2,

Caspase-3 – cysteine-aspartic acid protease-3, Notch1 – Neurogenic locus notch homolog protein 1.

Table S1: Differentially expressed mRNAs across time points post-injury.

Results of differential expression analysis at 3 hpi, 12 hpi, 24 hpi, 3 dpi, and 7 dpi. Each sheet corresponds to one time point. Columns include: baseMean (mean normalized counts), log2FoldChange, lfcSE (standard error of log2FC), stat (test statistic), pvalue, padj (adjusted p value, FDR), Ensembl gene ID, and symbol (gene symbol).

Table S2: Gene set enrichment analysis (GSEA) based on Gene Ontology terms

Significantly enriched biological processes, molecular functions, and cellular components identified from DEGs at each time point. Columns include ontology category (*ONTOLOGY*), GO *ID* and *Description*, *setSize*, *enrichmentScore*, *NES* (normalized enrichment score), *pvalue*, *p.adjust* (FDR), *qvalue*, *rank*, *leading_edge*, *core_enrichment*. Added columns: *time* (time point and direction, e.g., "3 hpi UP"), and *ratio* (number of genes contributing to enrichment relative to *setSize*).

Table S3: Over-representation analysis of curated gene sets.

ORA results of 118 gene sets compiled from 21 published studies, tested for overlap with DEGs from this study. Columns include: *ID*, *Description*, *GeneRatio* (DEG overlap/total DEGs), *BgRatio* (overlap/total background), *pvalue*, *p.adjust*, *qvalue*, *geneID*, *time* (time point and direction), and *ratio* (number of overlapping genes).

Table S4: Proteomics dataset (mass spectrometry).

Normalized and imputed protein abundance values. Columns represent samples, and rows represent individual proteins. The first column (*name*) indicates the protein symbol.

Table S5: WGCNA module eigengene characterization.

Summary of co-expression modules and enrichment results. Five spreadsheets are provided:

A. **Modules info** – module size and identifier (*ModuleColors*, number of genes, *module_name*).

B. **Modules info2** – gene membership and cross-species annotation (Ensembl ID, *ModuleColors*, module eigengene correlations, gene symbols/IDs across rat and mouse).

C. **DEGs enrichment** – ORA of module genes against DEGs from Table S1 (*ID*, *Description*, *GeneRatio*, *BgRatio*, *pvalue*, *p.adjust*, *qvalue*, *geneID*, module, *ratio*).

D. **Cell-type est** – ORA of module genes against CNS cell-type marker genes (Milich et al., 2021 re-analysis).

E. **ORA** – ORA of modules with GO Biological Process terms, including grouped categories (*group*).

TableS6 Differentially expressed miRNAs across time points post-injury.

Results of differential expression analysis for miRNAs at 3 hpi, 12 hpi, 24 hpi, 3 dpi, and 7 dpi. Columns include: baseMean, log2FoldChange, lfcSE, stat, pvalue, padj, and mirnaid.

Table S7: Interactome of regulatory relationships.

Comprehensive list of predicted and observed interactions between different molecular layers.

Two spreadsheets are provided:

A. **miRNA–mRNA interactions** – calculated correlations between miRNA and mRNA expression (*miRNA*, *mRNA*, *pval*, *cor.coef*, *fdr*, *gene_symbol*, *ensembl_peptide_id*).

B. **mRNA–protein interactions** – calculated correlations between mRNA and protein expression (*mRNA*, *protein*, *pval*, *cor.coef*, *fdr*, *ensembl_peptide_id*).

Table S8. The list of the primary antibodies.

Antibodies name	Catalog number #	Molecular weight	Isotope	Dilution solution	Dilution
Annexin V	8555	30	Rabbit mAb	BSA	1:1000
Neuro D1	7019	49	Rabbit mAb	BSA	1:1000
NF-H	2836	180-220	Mouse mAb	BSA	1:800
Notch1	4380	120, 300	Rabbit mAb	BSA	1:1000
MAP2	4542	75, 82, 280	Rabbit mAb	BSA	1:1000
GFAP	12389	50	Rabbit mAb	BSA	1:400
Nogo-A	13401	180	Rabbit mAb	BSA	1:1000
Caspase-3	14220	35,19,17 kDa	Rabbit mAb	BSA	1:1000
β-Actin	A2228	42 kDa	Mouse mAb	TBST	1:2000
